# DSAVE: Detection of misclassified cells in single-cell RNA-Seq data

**DOI:** 10.1371/journal.pone.0243360

**Published:** 2020-12-03

**Authors:** Johan Gustafsson, Jonathan Robinson, Juan S. Inda-Díaz, Elias Björnson, Rebecka Jörnsten, Jens Nielsen

**Affiliations:** 1 Department of Biology and Biological Engineering, Chalmers University of Technology, Gothenburg, Sweden; 2 Wallenberg Center for Protein Research, Chalmers University of Technology, Gothenburg, Sweden; 3 Mathematical Sciences, University of Gothenburg and Chalmers University of Technology, Gothenburg, Sweden; 4 Department of Molecular and Clinical Medicine, Wallenberg Laboratory for Cardiovascular and Metabolic Research, University of Gothenburg, Gothenburg, Sweden; 5 BioInnovation Institute, Copenhagen, Denmark; Indraprastha Institute of Information Technology Delhi, INDIA

## Abstract

Single-cell RNA sequencing has become a valuable tool for investigating cell types in complex tissues, where clustering of cells enables the identification and comparison of cell populations. Although many studies have sought to develop and compare different clustering approaches, a deeper investigation into the properties of the resulting populations is lacking. Specifically, the presence of misclassified cells can influence downstream analyses, highlighting the need to assess subpopulation purity and to detect such cells. We developed DSAVE (Down-SAmpling based Variation Estimation), a method to evaluate the purity of single-cell transcriptome clusters and to identify misclassified cells. The method utilizes down-sampling to eliminate differences in sampling noise and uses a log-likelihood based metric to help identify misclassified cells. In addition, DSAVE estimates the number of cells needed in a population to achieve a stable average gene expression profile within a certain gene expression range. We show that DSAVE can be used to find potentially misclassified cells that are not detectable by similar tools and reveal the cause of their divergence from the other cells, such as differing cell state or cell type. With the growing use of single-cell RNA-seq, we foresee that DSAVE will be an increasingly useful tool for comparing and purifying subpopulations in single-cell RNA-Seq datasets.

## Introduction

All cells in the human body are unique—no two cells have exactly the same transcriptional profile. Recent advances in single-cell RNA sequencing have enabled examination of the heterogeneity among individual cells, including data from several hundreds of thousands of cells in the same experiment [1–3]. A difficulty with single-cell RNA-Seq data is its high cell-to-cell variation due to low sampling depth and transcriptional bursting [4], which makes it challenging to extract useful information when comparing the transcriptomes of individual cells. To remedy this, a variety of cell clustering algorithms have been developed [5–7], which reduce variation by enabling comparisons between cell populations (defined here as collections of single cells with associated count matrices) instead of individual cells.

The purpose of clustering is to create cell populations based on biological traits, such as cell type or different cell states. Despite the variety of clustering algorithms available, misclassification of cells, where some cells assigned to a cluster exhibit a greater biological difference from the others, is still a large problem [6–8]. The difficulty increases when trying to separate more biologically similar cells, such as subsets of B cells, since in such cases the technical noise becomes relatively higher compared to the biological variation. It is naïve to expect that the outcome of clustering can be fully trusted without additional analysis, and in some cases purification (removal of cells that are deemed to not belong to the cluster). To our knowledge, two tools have previously been specialized for the detection of misclassified cells. scReClassify [9] is based on dimensionality reduction by PCA followed by a semi-supervised learning method to detect misclassified cells, while Jackstraw [10], based on dimensionality reduction and Jackstraw calculations, estimates the probability that a cell belongs to the assigned cluster.

In addition to misclassifications, the data for some cells may be of poor technical quality. There are many existing cell-wise quality control metrics for single-cell RNA-Seq data (e.g. number of unique molecular identifiers (UMIs), mitochondrial gene content, etc.), and a common approach is to discard cells based on a combination of these metrics [11, 12]. Choosing thresholds for these metrics is a balance between removing as many poor-quality cells as possible and not losing too much of the dataset. Although software packages for single-cell analysis such as Seurat [13] offer tools for finding these thresholds, it is difficult to prevent some low-quality cells from passing quality control.

The high technical variation in single-cell sequencing data poses a challenge for clustering as well as downstream analyses. The often-dominating source of technical variation is the sampling noise, which arises from the limited number of mRNA molecules and reads per cell, where two perfectly identical cells will appear different due to random and incomplete sampling of their transcriptome. We define sampling noise as the variation between cells drawn from a multinomial distribution, Mult(*n*, **p**), where *n* is the number of molecules sampled from a cell and **p** is a vector describing the probabilities of drawing molecules and is directly proportional to the mean expression of each gene in a cell population. This model has previously been used for describing sampling noise for UMI-based technologies [14], and has for example been used in MetaCell to group cells that are sampled from the same multinomial distribution [15]. However, this model does not hold for technologies that do not identify unique molecules, since several copies of the same molecule may be read, and therefore reads are not independent [16]. An important distinction between sampling noise and other types of technical variation is that the sampling noise approaches zero as the number of identical cells approaches infinity. Other technical sources of variation are not as easily remedied since they often introduce systematic biases into the data.

Measuring the variation in cell populations could be used to estimate cluster homogeneity; such a metric would ideally include all biological, but no technical, variation. In addition, variation measurements could potentially be used as a quality metric for datasets. In this case, the technical variation associated with sampling noise would be of interest, while the biological variation ideally should be excluded. Such ideal measurements are however challenging to perform. Correcting for technical factors including sampling noise is supported by packages such as scran [17], where the correlation between variation and gene expression is modeled as technical variation. Such transformed data could be used for estimating cluster homogeneity, but additional processing would be required. Furthermore, such a method is less suitable for estimating dataset quality, since it is not guaranteed to only remove sampling noise but also additional factors that vary with gene expression. It is also possible to measure the technical variation in single-cell RNA Sequencing experiments by adding non-biological spike-in genes in an equal amount to all cells, where all variation in those genes will be interpreted as technical [18, 19]. This strategy suffers from similar problems, in that the variation will be a mix of sampling noise and technical noise, and that any technical variation introduced before cell lysis (when spike-ins are usually added) is not captured.

Cell-type specific gene expression profiles (GEPs) are useful for advanced computational methods such as digital cytometry [20] and genome-scale modeling [21, 22]. Such GEPs can be created from single-cell RNA-Seq by pooling data from single cells, where the GEP is formed from the average expression of the cells [23]. Creating GEPs from clustered single-cell data is advantageous, since in contrast to cell sorting methods such as FACS [24] it allows for a more flexible approach in selecting cell populations. The number of cells pooled when creating a GEP is a critical factor for producing a representative expression for a cell population. Although quality increases with more cells, it is valuable to know how many cells are needed to reasonably represent a cell population due to high sequencing costs or limited sample material. In addition, selecting the pool size is a balance between reducing stochasticity from the limited number of cells and being precise in selecting cells representative of the population of interest. A third factor to consider is which genes are of interest. In general, to obtain a stable gene expression value, lowly expressed genes require more cells.

Here, we sought to address three problems in single-cell data: 1) determining a suitable pool size for generating cell-type specific GEPs; 2) estimating the purity of single-cell clusters; and 3) identifying misclassified or low-quality cells. In addition, we investigated if the quality of clustered single-cell data can be estimated from the cell-to-cell variation by identifying technical non-sampling variation. To address these problems, we developed DSAVE (Down-SAmpling based Variation Estimation), which includes methods for estimating cell population size, finding misclassified cells, and estimating the non-sampling cell-to-cell variation in cell populations. For the latter method, we introduce the concept BTM (Biological, Technical, and Misclassifications) variation, which represents all cell-to-cell variation within a cell population except sampling noise. The metric is comparable across cell types and datasets since it is independent from the often large and population-dependent sampling noise. We show that the BTM variation can be used as a relative measure for cluster purity, and demonstrate its utility in identifying datasets with high non-sampling technical variation, since the technical variation in such cases is much larger than its biological counterpart.

## Methods

### Data preparation

To enable verification of our methods, we downloaded the public single-cell RNA-Seq datasets listed in Table 1. Cell type classification was retrieved from the authors of the study in cases where it was not publicly available. Dataset access information is given in S1 Table.

**Table 1:** List of single-cell datasets used in this study.

ID	Description	Source
HCA CB	Umbilical cord blood PBMCs from the Human Cell Atlas; in total ~254,000 cells from 8 patients.	Li et al. [1], Rozenblatt-Rosen et al. [2].
BC	Immune cells from breast cancer patients; from tumor, healthy breast tissue, lymph node and blood. In total ~47,000 cells from 8 patients.	Azizi et al. [25].
LC	~39,000 cells from the tumor microenvironment of lung cancers and ~13,000 cells from adjacent healthy tissue. The cells originate from 5 patients.	Lambrechts et al. [26].
OC	~3,000 cells from ovarian cancer ascites, originating from 4 patients.	Schelker et al. [27].
LIVC	~5,000 T cells from the tumor microenvironment of liver cancer, originating from 6 patients. Unique molecular identifiers (UMIs) have not been used for this dataset.	Zheng C. et al. [28].
PBMC68k	~68,000 PBMCs from blood, one patient.	Zheng G.X.Y. et al. [3].
B10k	~10,000 FACS-sorted CD19+ B cells from blood, one patient.	Zheng G.X.Y. et al. [3].
CD4TMEM	~10,000 FACS-sorted CD4+/CD45RO+ Memory T Cells, one patient.	Zheng G.X.Y. et al. [3].
TCD8	~10,000 FACS-sorted CD8+ T cells from the blood of a single patient.	Chen et al. [29].
LIVC2	~66,000 cells from 5 liver cancer patients, from 5 different tissues.	Zhang et al. [30].

We furthermore downloaded 8 bulk RNA-Seq samples from the BLUEPRINT Epigenome Project [31]. The samples were taken from the project EGAD00001001173 and have the following sample IDs: S002EV11, S004M711, S007DD11, S007G711, S008H111, S009W411, S001FRB1, and S0041C11. The FASTQ files were first processed using kallisto [32] to produce gene counts (estimated counts produced by kallisto). The data was then normalized using TMM [33], and scaled to an average count of 10^6^ per sample.

### DSAVE overview

DSAVE supports three types of calculations. First, the *total cell pool variation estimation* measures the total variation between pools of cells drawn from a cell population as a function of pool size. This metric is useful for comparing variability both between pooled single-cell samples and to bulk samples to estimate the number of cells that are needed in a pool to achieve similar variability as in bulk. Second, the *BTM variation score* describes the overall BTM variation in a cell population. The variation score is comparable across cell populations, even from different datasets. Finally, the *cell-wise variation metric* is used for detecting cells that diverge from the mean cell population expression and identify which genes are primarily responsible for the divergence of such cells. In addition to these three calculations, DSAVE also supports investigation of variation per gene (S4 Note).

**Fig 1:**
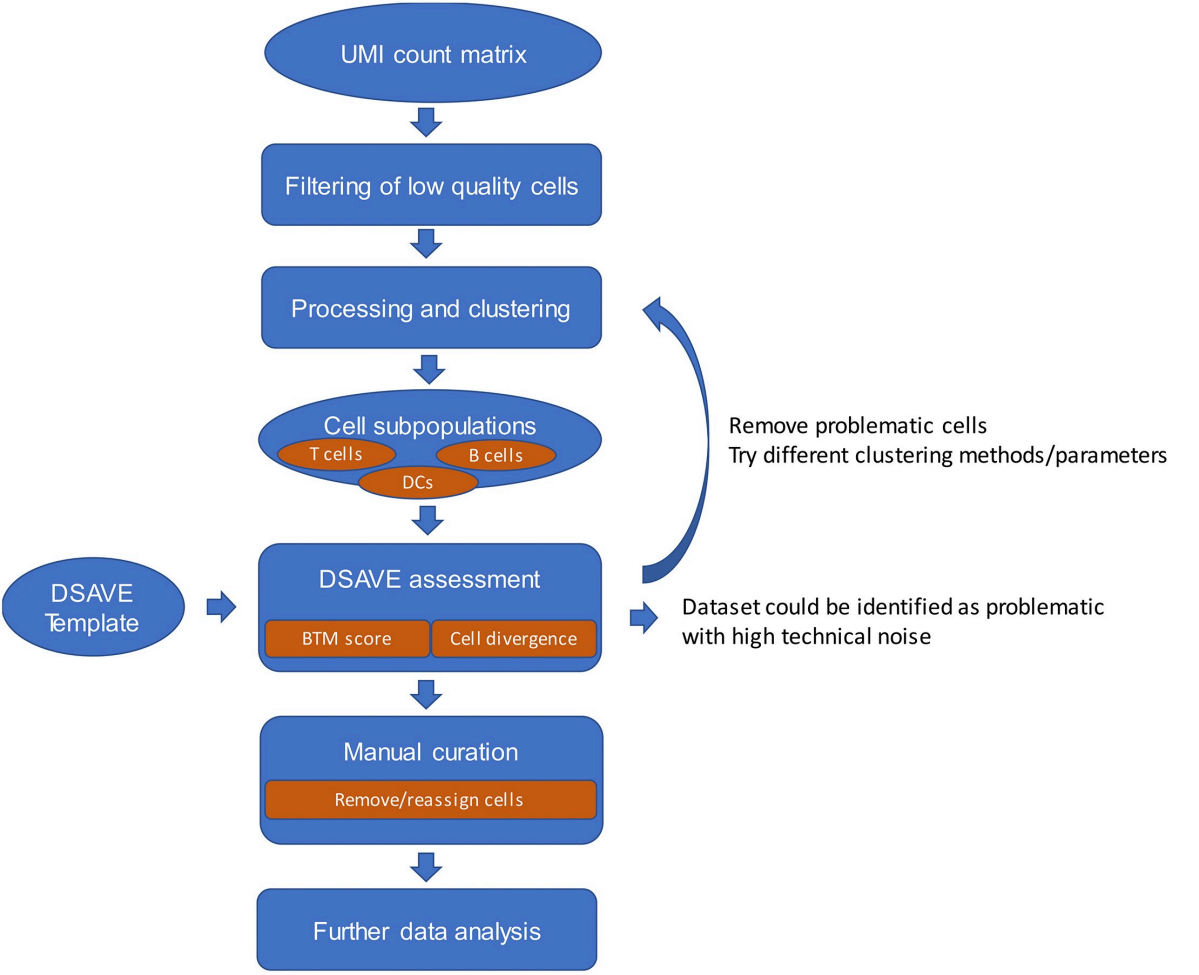
Typical use case for the DSAVE BTM variation score and DSAVE cell divergence. Ovals represent data while rounded rectangles represent data processing. The DSAVE BTM variation score and cell divergence are both applied to cell populations defined by clustering, using the original UMI count data in combination with cell clustering assignments. DSAVE allows for an iterative approach where the user can remove/reassign cells, experiment with clustering parameters, and assess the outcome, both in terms of total cell variation within the cluster (the BTM variation score) and detected misclassified cells. When the results are satisfactory the user can finalize the curation and proceed to further data analysis. The BTM variation score calculation requires a DSAVE template, which is explained further below in the methods section.

The typical use case for the DSAVE BTM variation score and cell divergence is depicted in Fig 1. Both metrics are applied to subpopulations of cells defined by clustering, which typically mainly contain cells of a single cell type. The purpose of DSAVE is to measure the homogeneity of such cell populations and detect misclassified cells. The DSAVE BTM variation score can be used as a measure of how diverse the cells in a cell population are. In general, clustering aims to put similar cells in the same cluster–it is thus possible to experiment with clustering parameters to minimize the BTM variation score. Furthermore, the cell divergence metric can be used to identify misclassified cells in a cell population. The divergence metric can be used to manually remove cells which are highly divergent or assign them to a different cluster, but also to identify which types of misclassified cells can be found in the cell population and remove all such cells manually using markers identified by the cell divergence output. Since the DSAVE BTM variation score is comparable across datasets, the metric can also be used for identifying datasets with high technical variation. This can be useful for example when selecting public datasets for analysis. The vignette in the DSAVE R package exemplifies further how DSAVE can be used, and additional advice on how to use DSAVE is available in S1 Note.

### DSAVE total cell pool variation estimation

The *total cell pool variation* is a measure of the total variation between cell pools sampled from a cell population. The input to the algorithm is a cell population from a single-cell RNA-Seq dataset, with either count or FPKM/RPKM/TPM data, and a gene expression range. The variation is calculated for 100 different pool sizes, chosen based on the total size of the cell population.

Quantifying the variation metric involves randomly selecting two pools without replacement from the total cell population, where the same cell may only be part of one pool. The variation metric *R* is then calculated as

$$R=\frac{1}{n_{genes}}\overset{n_{genes}}{\underset{i=1}{\sum }}\left|\log_{2}\left(\frac{expr_{1,i}+pc}{expr_{2,i}+pc}\right)\right|$$ (1)

where *expr*_*a*,*i*_ is the mean expression of gene *i* in pool *a*. The addition of the pseudo count *pc* is to avoid division by 0 and taking the logarithm of 0. The pseudo count can be adjusted depending on the desired weight to place on lowly-expressed genes; in this study we used pc = 0.05. To reduce stochasticity, an average metric *R*_*mean*_ is calculated for each pool size as the mean of *R* over 30 iterations of random pool sampling. The number 30 was chosen empirically based on its yield of a smooth curve and reasonable computation time.

Eight publicly available CD4+ T cell bulk RNA-Seq samples from BLUEPRINT were used to generate a comparable metric for typical bulk samples. The metric is calculated as the mean of all pairwise comparisons between the samples, calculated using the same formula as above, where *expr*_*a*,*i*_ is the expression of gene *i* in bulk sample *a*.

### DSAVE BTM variation score

#### Overview

**Fig 2:**
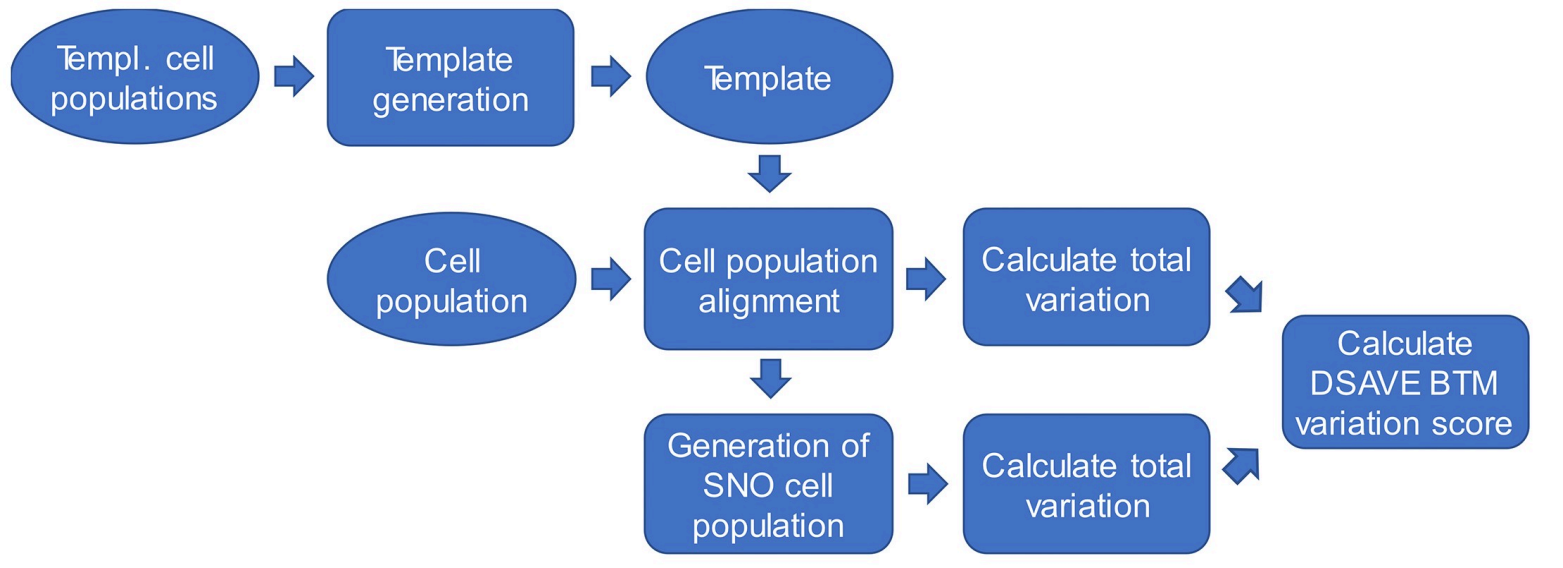
Overview of the calculation of the DSAVE variation score. Oval shapes represent data, while rounded rectangles represent calculations.

Fig 2 shows an overview of the calculation process used to estimate the *DSAVE BTM variation score*.

The inputs to the DSAVE BTM score calculation are a cell population from a single-cell RNA-Seq dataset with UMI count data and a DSAVE BTM variation score template. A template, which in many cases is supplied with the DSAVE package, can be created from a collection of cell populations together with parameters specifying the properties of the template, and is often compatible with many different types of cell populations. The template is needed to ensure that the BTM variation score is comparable between cell populations and is used in a process named cell population alignment. In this process cell populations are adapted in several ways, for example downsampled, to create aligned cell populations that have equal sampling noise. After cell population alignment, a SNO (Sampling Noise Only) cell population is generated from the aligned cell population by sampling from the mean gene expression profile of the population. This cell population is similar to its aligned counterpart, except that the only variation present is sampling noise. The total variation, based on the coefficient of variation (CV), is then calculated as a function of gene expression for both the aligned and SNO cell populations. The variation of the SNO cell population is then subtracted from the variation of the aligned cell population to obtain the difference between total variation and sampling noise at different levels of gene expression. This difference represents the BTM variation and is used to calculate the DSAVE variation score. The following sections describe these steps in more detail.

#### Template generation

One typical use case for the BTM variation score is to calculate the score for one cell population and compare it with the scores of cell populations from other datasets. To make this practical, it is convenient to pre-calculate the scores of the other datasets and include them with the software package. The DSAVE variation score is only comparable among different cell populations when using a common set of genes, the same number of cells, and identical distributions of counts per cell, which is matched in a procedure called alignment (described below). This property of the score makes it challenging to pre-calculate comparable values for cell populations. Some genes may not be present in a new cell population that is to be investigated, it may not have the same number of cells, and the distribution of counts may be such that it cannot be downsampled to match the distribution of the populations for which the score was precalculated. To solve this issue, a template is defined, which states the genes included, the number of cells to use, and/or the distribution of counts per cell to which the populations should be downsampled. The template parameters are selected to work for most datasets while still using enough data to produce a stable score calculation.

The template is created from a master cell population, a list of datasets for gene selection, the target number of cells (N), the target average counts per cell (C), and the upper and lower fractions of outliers to discard (explained in Calculation of total variation). The genes to use are chosen as the intersection of the genes available in the dataset list supplied. N cells are then randomly selected from the master cell population, and randomly downsampled to yield a count distribution per cell with an average count that equals C, thereby generating a cell count distribution to use for calculation of the DSAVE variation score. Several standard templates, which require different numbers of cells, were derived from several datasets and are provided with the DSAVE package along with precalculated scores for multiple cell populations. The vignette of the R package describes how to create a new template should the need arise.

#### Cell population alignment

The purpose of cell population alignment is to normalize all cell populations to achieve similar sampling noise while retaining their original BTM variation. The alignment procedure uses the information stated in a template to first randomly sample a fixed number of cells (as stated in the template) from the original population to include in the aligned counterpart. The second step is to discard all genes which are not included in the template. In the final step, all cells in the population are randomly downsampled to match the distribution of total reads per cell that is stated in the template (more details are provided in S1 Note). The output of the alignment is thus an aligned cell population, which has similar BTM variation as the original cell population, but a sampling noise matching that specified by the template and thereby all other aligned cell populations.

#### Generation of SNO cell populations

The purpose of generating a SNO cell population from an aligned cell population is to obtain a cell population with similar sampling noise but no additional sources of variation, meaning that all cells in the population are identical apart from the sampling noise. To achieve this, we simulate gene counts for each cell by randomly sampling reads from a multinomial distribution, Mult(*n*, **p**), where **p** is a vector of probabilities based on the cell population’s mean expression of the genes and n is the total number of counts for the cell. The probability *p*_*i*_ for each gene in the **p** vector is calculated as

$$p_{i}=\frac{E_{i}}{10^{6}}$$ (2)

where *E*_*i*_ is the gene expression in counts per million (CPM).

#### Calculation of total variation

The DSAVE total variation metric is based on the coefficient of variation (CV), calculated gene-wise over all cells in the population. The total variation metric *D*_*i*_ for gene *i* is calculated as

$$D_{i}=ln\left(CV_{i}+1\right)$$ (3)

where *CV*_*i*_ is the coefficient of variation for gene *i* over its gene expression values in CPM in all cells. The addition of the offset 1 is to ensure that *D*_*i*_ is always positive. The reason for log transforming the CV is that it creates a metric for the BTM variation that is relatively constant over the full gene expression range. *CV*_*i*_ is calculated as

$$CV_{i}=\left(SD_{i}\right)/\left(M_{i}+0.05\right),$$ (4)

where *CV*_*i*_, *SD*_*i*_ and *M*_*i*_ are the CV, standard deviation, and mean of the gene expression. A value 0.05 is added to avoid dividing by a very small number or zero, minimizing the impact of very lowly expressed genes. The method also supports calculation of variation using log transformed data, which reduces the impact of outliers (S1 Note). The low value of 0.05 used for division was chosen to achieve a higher sensitivity for lowly expressed genes, for which a higher value of e.g. 1 would give a smaller CV. The two alternatives for calculating the variation were compared, showing only small differences in DSAVE variation score output (S1A Fig).

**Fig 3:**
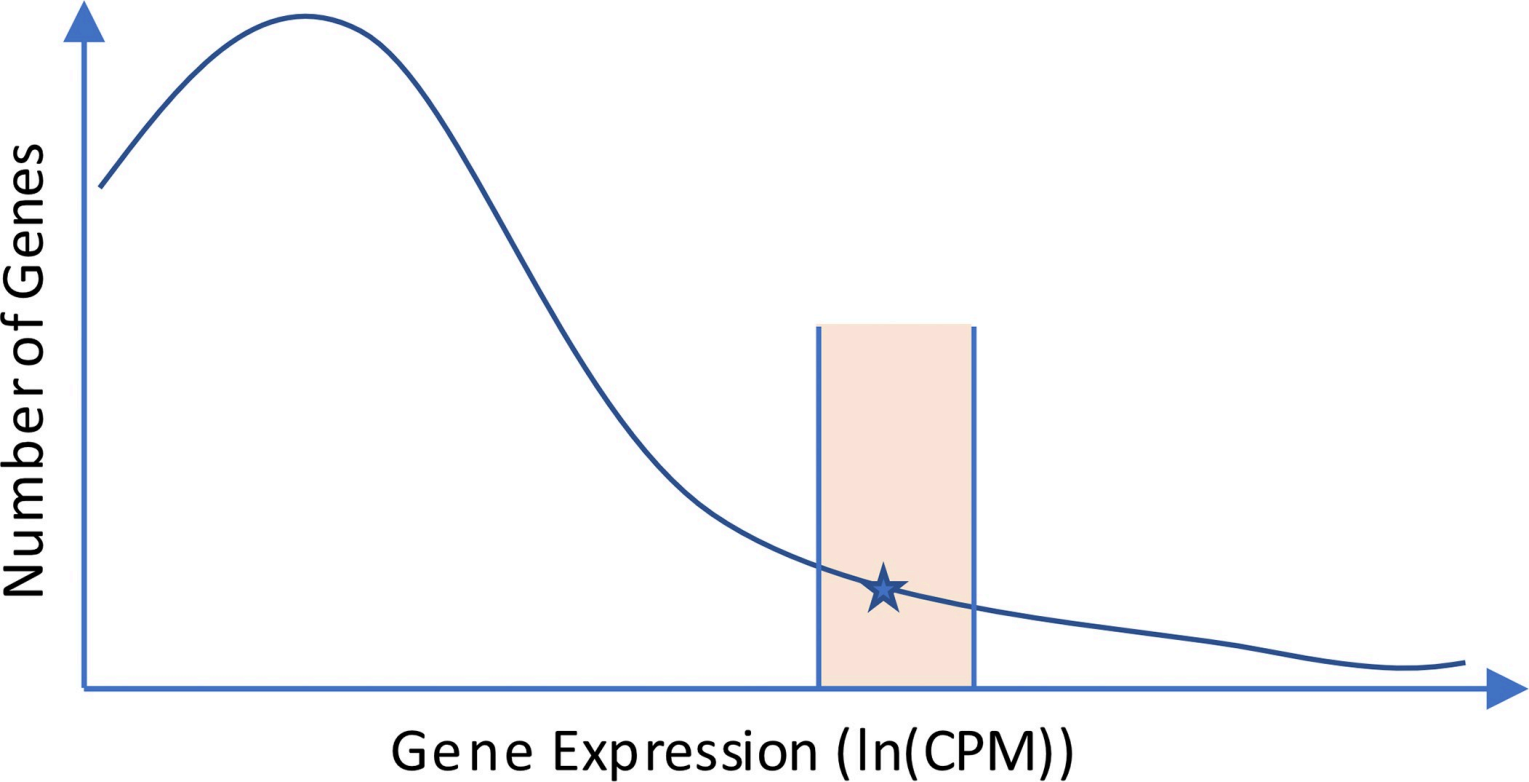
Schematic visualization of how the variation per gene expression range is calculated. 1000 points are logarithmically distributed between 10 and 1000 CPM. For each point, a range is determined based on the template cell population. The range (shaded region) for a point (the star in the figure) is defined to cover 500 genes such that the geometric mean of their expression lies as close to the point as possible. The bounds 10–1000 CPM were determined empirically. For genes below 10 CPM, the spread in variation was generally high in comparison to the difference in variation between the aligned and SNO cell populations. Above 1000 CPM, the low frequency of genes resulted in large expression ranges that were no longer appropriate to represent with a single CPM value. The range width of 100 genes was also determined empirically; 500 genes produces a stable variation metric while still maintaining a reasonable representation of the CV distribution.

To make the total variation metric comparable across cell populations, it is not suitable to compare the variation gene-wise since the expression of a gene can vary between cell populations. Likewise, calculating the mean over all genes would introduce a bias if the gene expression distribution differs between cell populations, since the BTM variation is not completely constant over the gene expression range. To minimize the impact of such differences, the variation is instead calculated separately for 1,000 partially overlapping ranges of gene expression. The ranges are selected as described in *Fig* 3 and are precalculated during template generation and stored in the template. A vector **D**_**r**_ of total variation values for all ranges is then calculated, where the total variation for each range value is calculated as the mean total variation of all genes within the range. The geometric mean expression of the genes within each range is also stored as a vector **E**_**r**_ for later use. Geometric mean is used instead of arithmetic mean because the gene expression values are not log transformed.

To reduce the effect of outlier genes on the variation score, we also remove the most and least variable genes (see S1 Note for details). The outlier thresholds specifying the fraction of the most and least variable genes to discard are included in the template.

#### DSAVE BTM variation score calculation

The DSAVE *BTM variation score* is calculated from the total variation of the aligned and SNO cell populations and is a metric describing the BTM variation in a cell population. For each of the two populations, a continuous function *f(e)* is generated by linear interpolation between values in the total variation vector **D**_**r**_ at points along the geometric mean expression vector **E**_**r**_. *f(e)* thus represents the mean total variation at gene expression *e*. The variation per gene expression is then sampled from *f(e)* at *k* = 1000 logarithmically distributed points between 10 and 1000 CPM for both the aligned and SNO cell populations, where the expression at point *j* is denoted *e*_*j*_. At each point, the variation of the SNO cell population is subtracted from the variation of the aligned cell population to yield a difference between the total variation and the sampling noise as a function of gene expression. To reduce stochasticity, the values are calculated as the mean of *n*_*it*_ = 15 iterations with different cell subpopulations sampled from the whole cell population, where the number of iterations were determined empirically (S1B Fig). This yields unique variants of *f(e)* for each iteration, which are denoted *f*_*a*,*i*_*(e)* and *f*_*SNO*,*i*_*(e)* for the aligned and SNO cell populations, respectively, at iteration *i*. The DSAVE BTM variation score (*DVS)* is then calculated as the mean of the differences between *f*_*a*,*i*_ *and f*_*SNO*,*i*_ across all points and iterations, according to:

$$DVS=\frac{\sum_{i=1}^{n_{it}}\sum_{j=1}^{k}f_{a,i}\left(e_{j}\right)-f_{SNO,i}\left(e_{j}\right)}{k\cdot n_{it}}$$ (5)

We note that that the DSAVE variation score is stochastic in nature and does not yield identical scores for repeated runs of the same data, although the scores are similar.

### DSAVE cell-wise variation metric

To identify cell outliers in a cell population, we determine for each cell the probability of measuring the observed count distribution, assuming that we sample from the mean gene expression profile of the cell population. To avoid multiplication of many small numbers, we calculate the log-likelihood instead of probability. To make the metric comparable between cells, we first down-sample all cells to the same number, *S*, of counts. Cells with fewer counts than *S* are discarded and will have an undefined log-likelihood. The probability *p*_*i*_ that a count sampled from the mean gene expression profile of the cell population belongs to gene *i* is calculated as

$$p_{i}=\frac{E_{i}}{\sum_{j}E_{j}}$$ (6)

where *E*_*x*_ is the mean expression (CPM) of gene *x* in the cell population. The probability *p*_*o*_ to get the observed counts vector **x** (over all genes) is then calculated as the probability density function of Mult(*n*, **p**) at the value **x**, where Mult is the multinomial distribution, *n* is the counts observed for the cell, and **p** a vector of all *p*_*i*_. The log likelihood, *L*, is then calculated as the natural logarithm of *p*_*o*_.

The log-likelihood is negative, where lower (more negative) values correspond to a larger difference between a cell and the mean gene expression of the cell population. We define the *cell divergence* as the negative log likelihood, yielding a positive value that increases with decreasing *p*_*0*_. To reduce stochasticity from down-sampling, the divergence is calculated 15 times, and the median of those values is used for each cell. The cell divergence cannot be compared between cell populations, since it depends on the gene expression profile and the number of counts to which the cells are down-sampled. The number 15 was determined empirically to yield a good correlation between runs (S1C Fig).

To investigate the cause of divergence for individual cells, we also introduced a log-likelihood metric per gene for each cell, the *gene-wise cell divergence*. The metric represents the probability p_o,i_ of getting the observed count for gene *i* by sampling from the average gene expression of the population. This metric is calculated from the probability density function of B_i_(*n*, *p*_*i*_), where *B*_*i*_ is a binomial distribution for gene *i*, *n* is the number of counts for the cell, and *p*_*i*_ is the probability that a randomly selected molecule belongs to the gene, as defined earlier. The log likelihood for each gene, *L*_*i*_, is calculated as the natural logarithm of *p*_*o*,*i*_. The gene-wise cell divergence is then defined as the negative log likelihood, *-L*_*i*_.

### Statistical analysis

To test the statistical significance of the reduction of BTM variation from removing divergent cells, we removed an equal number of randomly selected cells and recalculated the DSAVE variation score. The procedure was repeated for 1000 iterations and we used a one-sided one-sample t-test with the null hypothesis that removing a randomly selected set of cells yields an equally large reduction in BTM variation as when the divergent cells are removed.

### Relative importance analysis

The purpose of the relative importance analysis was to determine which factors are most important for explaining the differences in variation between different cell populations. We used the R package *relaimpo* [34] (v. 2.2–3) for the relative importance analysis and used the analysis type *lmg*. We created a DSAVE template using only 1000 cells, which enabled us to gather 51 cell populations from four datasets, five tissues (where umbilical cord blood and venous blood were considered the same tissue) and five cell types. The DSAVE BTM variation score was calculated for all samples and used as dependent variable in the analysis. We then created a design matrix of Boolean variables for the samples, where each variable represented either a dataset, a cell type or a tissue. One variable of each type was omitted and merged into an intercept (the HCA CB dataset, tissue blood and cell type T cell); all other factors therefore represent how they explain any differences in score from the samples matching the intercept. For the LC dataset, the intercept was represented by healthy tissue instead of blood, due to the lack of blood samples. In practice, this means that the LC dataset factor also includes any differences in BTM variation between blood and healthy lung tissue.

Since all factors were not represented in an equal number of samples, we scaled the output importance for each factor f with *1/n*_*f*, *samp*_, where *n*_*f*, *samp*_ is the number of samples for which each factor was involved, followed by a renormalization to a total sum of 100 for all factors. This scaling is motivated by that the lmg method is based on R^2^ reduction, which should be proportional to the number of points a factor is involved in.

This methodology could be used for dissecting the importance of other factors for explaining the variation in gene expression. The R code for the relative importance analysis is therefore made available in GitHub together with the MATLAB code, and can be accessed as described in S1 Note.

### Software

The DSAVE algorithms were originally implemented in MATLAB R2018b. The relative importance analysis was run in R, version 3.5.1. We have also implemented DSAVE as an R package to increase its accessibility. The DSAVE R package can be used independently, and is also compatible with Seurat [13], which is a common tool for single-cell data analysis. Information on how to access the software is available in S1 Note. An analysis on expected computational time and memory requirements is also supplied (S2 Note).

## Results

### Data preparation

We collected 9 publicly available single-cell RNA-Seq datasets from human samples and 8 bulk RNA-Seq samples of human CD4+ T cells from blood. From these datasets we generated all cell populations described throughout the results section. S2 Table describes the cell populations used for each figure in more detail.

### The cell pool size needed for stable gene expression

**Fig 4:**
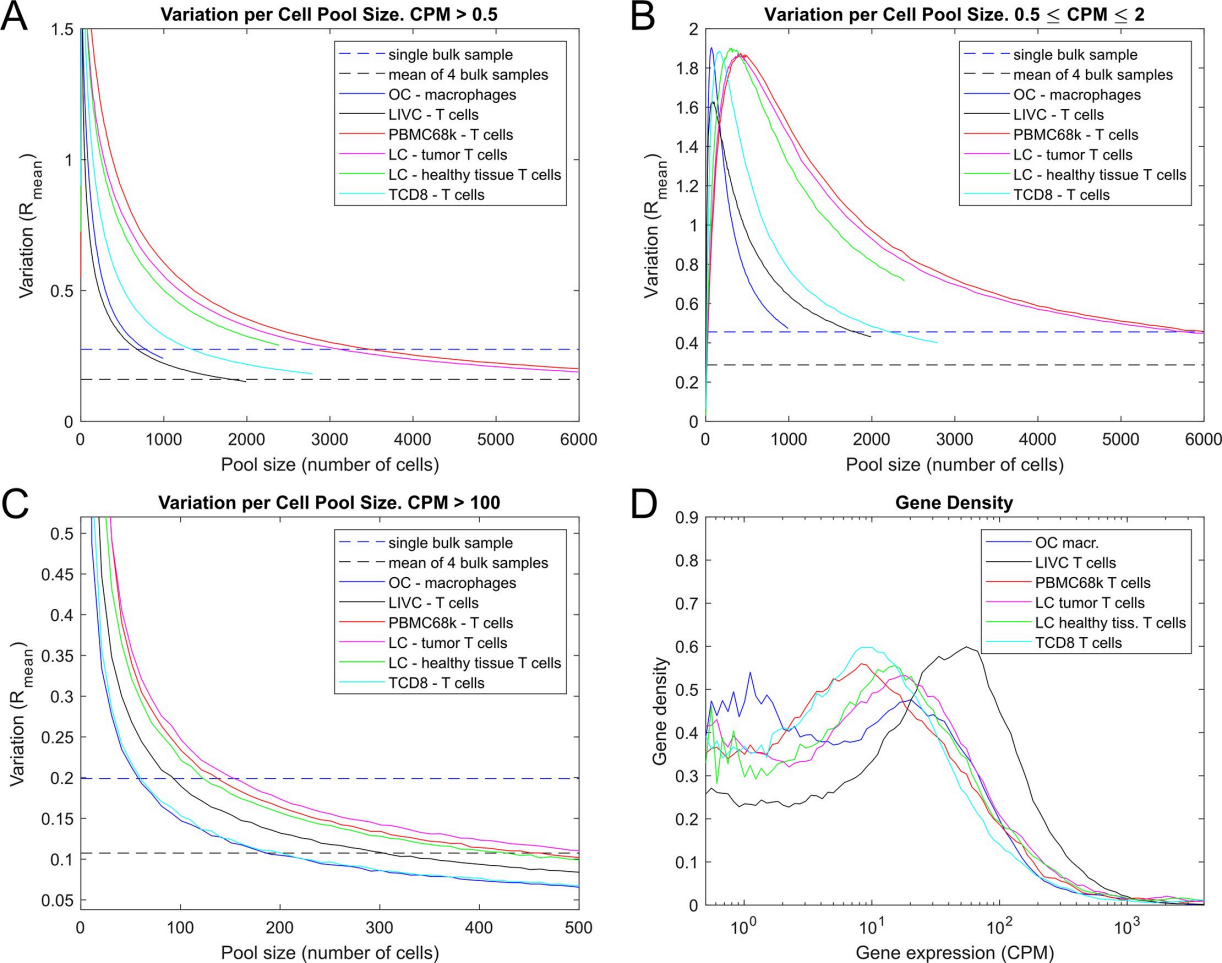
Cell pool size needed for stable average gene expression. A-C. DSAVE total cell pool variation estimation of 6 cell populations for different gene expression ranges, compared with the average variation of a bulk sample and the average variation of the mean of 4 bulk samples. D. Gene expression density in log_10_ scale for 6 cell populations from different datasets in the range of 0.5–4000 CPM. The graph shows that the highly expressed genes are few in comparison to the lowly expressed genes, and that this distribution varies between cell populations.

We began by investigating how the variation in the mean expression of cell pools depends on pool size. We calculated the DSAVE total cell pool variation for six cell populations with varying numbers of reads per cell. Fig 4A shows the variation as a function of cell pool size for different cell populations. In general, populations with a higher number of reads per cell exhibit a more rapid decrease in variation with increasing pool size, which can be attributed to a faster reduction in sampling noise.

To investigate how the variation depends on gene expression, we calculated the total variation for lowly (0.5 ≤ CPM ≤ 2) and highly (CPM > 100) expressed genes separately (Fig 4B and 4C). Lowly expressed genes exhibit relatively higher variability in single-cell datasets as compared to bulk data. The reason for this could be that the total number of molecules for lowly expressed genes are still few for single-cell data even though many cells are pooled, whereas a bulk sample is likely based on more cells, yielding more molecules and a more stable expression between measurements. For highly expressed genes, sampling has a lower impact on variability; other sources of variation thus become more important at small pool sizes.

We also investigated the gene count distribution for different cell populations to understand how differences in this distribution contribute to the total variation. Fig 4D shows that highly expressed genes are few in comparison to lowly expressed genes, implying that the curves in Fig 3A are dominated by the lowly expressed genes. The plot also shows that the gene expression distributions differ between cell populations, indicating that a simple mean of the variation over all genes will not yield an informative comparison between datasets, even if the number of reads per cell are the same.

### Evaluating the non-sampling variation of cell populations

When generating GEPs from single-cell data, it is important to minimize cell misclassifications and technical variation. It would therefore be valuable to measure these properties, since it would enable selection of datasets with small technical variation and detection of mixed cell populations. Although the BTM variation of a cell population is not a direct measure of these properties, it is useful for estimating the reliability of pooled gene expression profiles, since both misclassifications and technical effects will increase the BTM variation.

**Fig 5:**
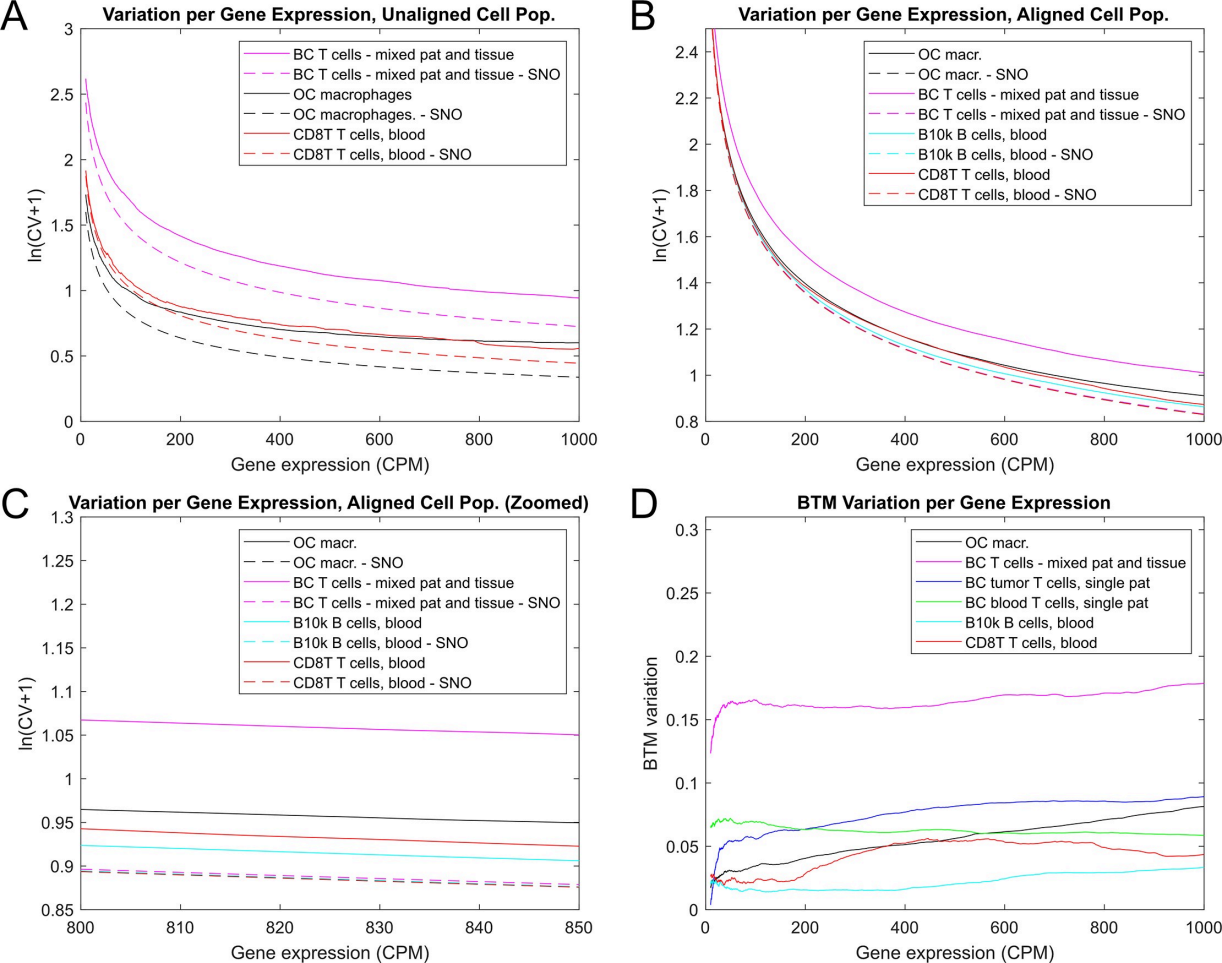
Investigation of BTM variation. A. Variation as a function of gene expression for 3 cell populations and their SNO counterparts. The cell populations have not been aligned. B, C. Variation as a function of gene expression for 4 aligned cell populations and their SNO counterparts. All SNO cell populations now have virtually identical variation, meaning that any difference in total variation between aligned cell populations corresponds to the difference in BTM variation. D. The difference between the variation for the observed and SNO cell population as a function of gene expression. These curves represent the BTM variation.

To quantify the BTM variation, we generated SNO (Sampling Noise Only) cell populations from the observed cell populations by randomly sampling the same number of reads from the mean expression profile of the observed cell population. Fig 5A shows the cell-to-cell variation for three datasets and their SNO counterparts. The BTM variation in this plot is represented by the y-distance between the curves for the original and SNO cell population. Although this representation provides some insight into which dataset exhibits the highest BTM variation, it is difficult to directly compare between cell populations.

The cell populations were aligned to a template to obtain populations with nearly identical sampling noise. This involved down-sampling and limiting the analysis to the set of shared genes (see Methods and S1 Note). To make the datasets comparable, it is also important to process the variation as a function of gene expression instead of pool size, since some cell populations may harbor more lowly expressed genes than others, especially across different cell types. Fig 5B and 5C shows four aligned cell populations and their SNO counterparts. The SNO datasets have virtually identical variation curves, and thus the variation curves for the aligned cell populations are directly comparable with each other as a metric for BTM variation. In Fig 5D, the difference between the aligned and SNO curves represent the BTM variation.

**Fig 6:**
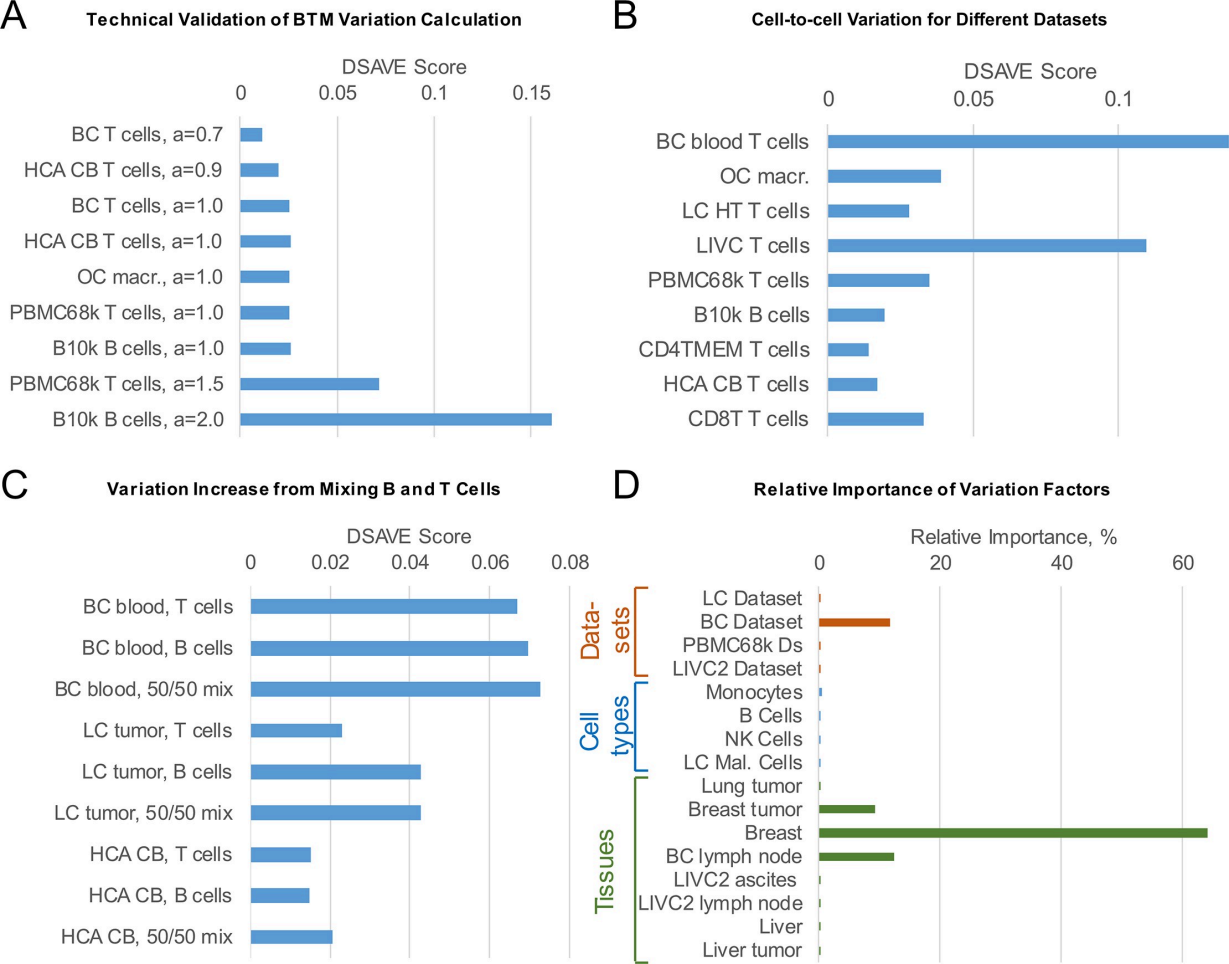
Evaluation of the DSAVE variation score. A. Technical validation of the DSAVE variation score. All cell populations were generated in a similar fashion as the SNO cell population, except the probabilities for each gene was multiplied by a noise factor f. The noise factor was calculated as f = 2N*a, where N is a standard normal distribution and a is a positive parameter that describes the magnitude of the noise. The probabilities are then normalized to a sum of 1. The figure shows an increasing score with increasing BTM variation, and demonstrates that the score is similar when the same noise level is applied, regardless of cell type or number of reads. B. BTM variation (DSAVE Score) for different datasets. C. Comparison between cell populations with 50% B cells and 50% T cells, and their pure counterparts, for a single patient. A specialized template with 1346 cells was used here due to small cell population sizes. D. Relative importance of variation factors calculated from 5 datasets. The graph shows which factors (dataset, cell type, and tissue of origin; indicated by red, blue, and green bars, respectively) can explain differences in the DSAVE variation score between cell populations.

To facilitate comparison of BTM variation across cell populations, we represent the difference in variation between the aligned cell population and its SNO counterpart as a single value called the DSAVE BTM variation score. We performed a technical evaluation of the DSAVE BTM variation score, shown in Fig 6A. We generated 9 SNO cell populations with added multiplicative noise from different datasets and different numbers of counts per cell. The results show that the metric yields higher values for cell populations with more added noise, and similar values for cell populations with the same added noise but different cell type or number of reads per cell.

We further investigated how the DSAVE variation score varies for a collection of cell populations from different public datasets, and found that the BTM variation differs substantially between them (Fig 6B). For all cell populations except the population from the LIVC dataset, molecules were tagged with a unique molecular identifier (UMI), which allows for removal of amplification bias. The relatively high variation score for the LIVC dataset could be partly explained by this difference in experimental procedure, and the fact that the multinomial sampling noise model assumes UMI counts, not raw counts [16].

To evaluate the ability of the DSAVE variation score to determine the quality of the cell type classification, we created mixes of B and T cells and compared their BTM variation to that of the pure cell populations. Fig 6C shows that a mix of B and T cells increases BTM variation. We also investigated the effect of mixing different proportions of cell types. Surprisingly, a mixed population containing more monocytes than T cells yielded a higher DSAVE variation score than a mix containing equal proportions of T cells and monocytes (S2A Fig). In addition, we evaluated the impact on the DSAVE variation score of mixing cells of the same cell type from different patients, indicating an increase in score for mixed populations (S2B Fig). This can likely be attributed to a combination of technical batch effects and biological variation between patients.

We then performed a relative importance analysis using the DSAVE variation score as the dependent variable (Fig 6D) to gain a better understanding of whether the BTM variation is technical (including misclassifications) or biological in nature. The covariates were all categorical and implemented as Boolean dummy variables, representing different properties of the cell populations. We included 68 cell populations from 5 datasets in the analysis, using a modified DSAVE template with only 1000 cells to calculate the score which allowed the inclusion of more populations. The analysis was based on the assumption that the biological variation for the same cell type in the same environment (blood, in this case) should be the same, regardless of dataset. The results show that in general the difference in BTM variation between cell types, which we interpret here as purely biological, seems to be small. Important factors contributing to BTM variation are dataset and tissue of origin, where contributions associated with differences in dataset are assumed to be primarily technical, particularly in cases where the differences are relatively large. The contribution associated with difference in tissue of origin is more difficult to interpret; both biological and technical variation is possible, where the technical variation could be attributed to different cell extraction protocols and challenges for different tissues. There is however a very strong correlation between tissue and dataset here; the variation for tissues is relatively small in all cases except for the tissues originating from the BC dataset, suggesting that there is a systematic technical difference between the datasets.

We also performed the relative importance analysis without the breast cancer dataset to see if technical factors still explained most of the differences in variation (S2C Fig). In this case, the PBMC68k dataset explained more than twice as much as any other factor, demonstrating that technical factors are likely the dominating contributor to BTM variation for several datasets. Our conclusion from this analysis, together with the fact that a mix of cell types gives a modest increase in variation, is that if the BTM variation is large for a population with cells of the same cell type, it is most likely technical in nature.

We conclude that there are two ways to use the BTM variation score: 1) to compare the BTM variation of different datasets, where a large variation may indicate a technical problem with either the dataset or the clustering method applied, and 2) to compare different ways of dividing a collection of cells into populations. For the latter, the DSAVE score should be calculated without removing outlier genes, since they are likely differently expressed in misclassified cells.

### Identifying misclassified and low-quality cells

To enable identification of misclassified and low-quality cells, we formulated a cell-wise variation metric, the *divergence*. The metric is based on the log-likelihood of getting the observed expression profile of each cell when sampling from the mean expression profile of the population (see Methods and S1 Note). The lower the divergence, the more likely it is to obtain the observed count distribution for the cell by sampling from the mean expression distribution of the cell population, indicating how similar a certain cell is to the other cells in the population.

**Fig 7:**
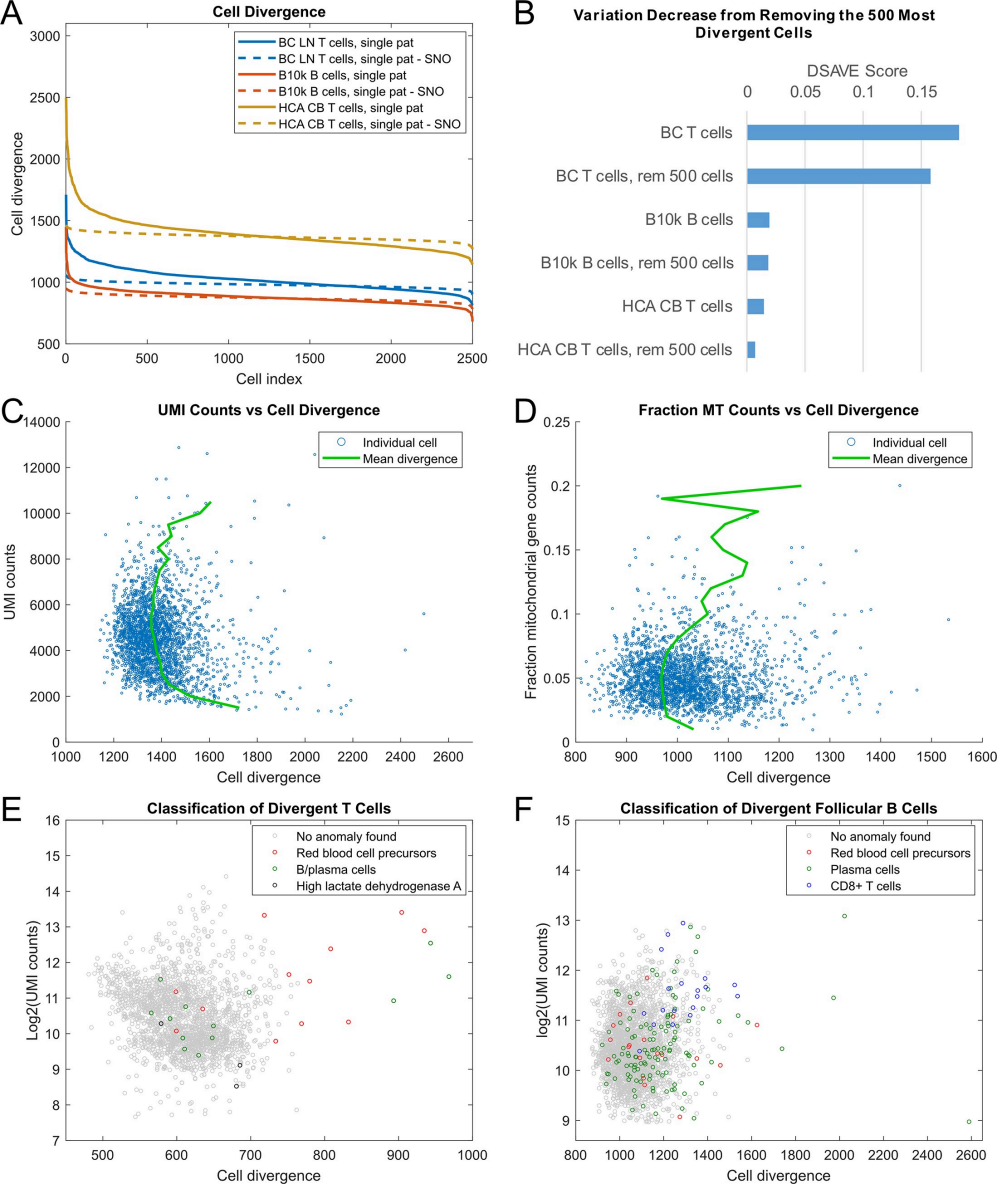
Evaluation of the DSAVE cell-wise variation metric. A. The distribution of cell divergence for the cells in three cell populations compared to their SNO counterparts. The cells are sorted by cell divergence. B. Decrease in variation upon removing the 500 most divergent cells from each dataset. C. UMI counts vs cell divergence for T cells from the HCA CB dataset. D. Fraction of counts belonging to mitochondrial genes vs cell divergence for T cells from the BC cell population. E, F. The divergence of T cells (E) and follicular B cells (F) from the LC dataset, showing potentially misclassified cells.

First, we investigated the difference in divergence between observed cell populations and their SNO counterparts (Fig 7A). The curves are not comparable between cell populations, only pairwise between a cell population and its respective SNO. The difference in divergence between cells in the original cell populations is larger compared to that of the SNO, and there appear to be outlier cells in all datasets.

Second, we investigated the effect of removing divergent cells from the population (Fig 7B). The results show a decreased BTM variation for the BC and HCA CB T cells, while the BTM score for the B10k dataset showed little change. The small change in the B10k dataset is likely because this cell population consists of FACS-sorted B cells, where few misclassifications are expected. However, implementing such a cutoff does not guarantee that the cells removed are only those of a different cell type or low quality, but can also remove cells that exhibit a greater difference from the population due to other sources of variability, such as sampling.

In many single-cell protocols each molecule is tagged with a unique molecular identifier, which allows for removal of amplification bias. A common quality control step in UMI-based single-cell analysis is to discard cells with low or high UMI counts [35]; we therefore investigated the relationship between cell divergence and UMI counts of each cell (Fig 7C). The plot shows that the dataset quality would probably benefit from an elevated lower threshold on UMI counts, since the cells begin to exhibit a sharp increase in cell divergence below 2500 counts. Likewise, we investigated the fraction of counts attributed to mitochondrial genes (Fig 7D), as high mitochondrial gene content has been shown to be associated with low quality cells [36]. The mitochondrial genes were excluded from the divergence calculation in this case to prevent the mitochondrial fraction from biasing the divergence. Cells with high mitochondrial gene content were more divergent, and the dataset would likely benefit from their removal. The relationship between number of detected genes and divergence was also investigated, suggesting a higher divergence for cells with more genes (S2D Fig). Such cells could potentially either represent doublets (i.e. droplets containing more than one cell) or cells with a larger transcriptome, for example highly activated cells, which also likely diverges in relative transcriptional profile across genes. However, when discarding cells, it is important to note that divergence is not necessarily a technical artefact and may be of biological interest.

Next, we investigated individual divergent cells to try to explain their high divergence. We used the *gene-wise cell divergence* (Methods) to first identify genes that largely contributed to the divergence of individual cells, by sorting the genes by the minimum value of their gene-wise cell divergence. Among the 40 genes with the lowest values, we found three groups of genes worth noting. Fig 7E shows the cell divergence for a population of T cells from the LC dataset. Some cells had very high expression (up to several thousand UMI counts) of the genes HBB, HBA1 and HBA2, which are all part of the hemoglobin complex. The LC dataset contains a cluster of red blood cell precursors (nucleated red blood cells, NRBC), and these results strongly suggest that the cells identified are either misclassified NRBCs or doublets (a mix of multiple cells), containing at least one NRBC. A second group of cells exhibited very high expression of genes belonging to the immunoglobin complex, suggesting either misclassification or doublets of B or plasma cells. A third group had high expression of lactate dehydrogenase A. This suggests that those cells are activated cytotoxic T cells, since activated T cells are known to increase aerobic glycolysis to maximize energy production [37]. The divergence of those T cells can thus probably not be explained by misclassifications or doublets, highlighting the fact that the divergence can also be used for finding interesting differences in biological behavior or state within cell populations. Interestingly, PCA and UMAP are not able to identify any of these misclassified cells (S2E Fig, S3 Note).

To determine if misclassification is a larger problem when trying to separate more similar cells into clusters, we investigated the population of follicular B cells in the LC dataset (Fig 7F). We initially used the same strategy as for the T cells and found a group of NRBCs and a large group of potential plasma cells with very high immunoglobin gene expression. It is unclear if the authors intended plasma cells to be included in this population or not, but removing them would most likely yield a more uniform population. We then investigated the remaining most diverging cells individually, by first sorting the genes for each cell based on the gene-wise cell divergence. For one cell, the gene GZMB, normally expressed in cytotoxic T cells and NK cells, was among the top genes. The cell also expressed CD8A, suggesting the cell to be a CD8+ T cell. We searched the population for more cells expressing CD8A and found a group of potential CD8+ T cells (UMI counts > 1).

Finally, we evaluated the ability of the DSAVE variation score to distinguish between the cell populations before and after removal of misclassified cells. We removed 25 NRBC and plasma cells from the T-cell population, resulting in a decrease in BTM variation from 0.1387 to 0.1353 (p < 10^−10^, one-sample t-test, comparing against the null hypothesis that removing randomly selected cells would yield the same decrease in BTM variation), which is a substantially larger difference than the uncertainty of the metric (S2D Fig). Interestingly, this subset of T cells exhibits a much larger BTM variation than the whole T cell population from this dataset, suggesting large variability in BTM variation within the dataset. Likewise, removing 156 NRBC, plasma cells and cytotoxic T cells reduced the BTM variation score from 0.0311 to 0.0280 (p < 10^−10^). These results show that the BTM variation score is useful for estimating cluster purity. The score in this case was calculated using a modified template, which used 1900 cells and discarded no outlier genes. The reason for this is that one of the cell populations had fewer than 2000 cells after outlier removal and that removal of gene outliers leads to less emphasis on misclassified cells in the score calculation, which is not desirable when evaluating cluster purity.

To investigate the utility of DSAVE, we evaluated the performance in finding misclassified cells compared to the tools Jackstraw and scReClassify. Since no ground truth is available for biological datasets, we detected misclassified cells with DSAVE (which we manually verified to be misclassified) to see if the same cells could be found using the other tools. While the other tools found many other potentially misclassified cells, they only found a small portion of the cells identified by DSAVE. This is likely because these tools operate on principal components, similar to the clustering, while DSAVE does not. (S3 Note)

To simplify analyses such as these, we have implemented an R function displaying an interactive map with all cells in a population, where hovering over a cell with the mouse displays the 5 most divergent genes and their divergence value (S3 Fig).

## Discussion

Single-cell RNA-Seq enables post-experimental formation of cell populations using clustering methods. However, the limited number of molecules in each cell combined with technical limitations of single-cell RNA-Seq and clustering methods introduce errors that may adversely affect downstream analyses. Although much effort has been spent on developing clustering methods, there is still a need for follow-up analysis and correction, since cell misclassifications are common. Minimizing such errors is essential to produce reliable output from analyses based on these generated cell populations, both indirectly to reduce the unwanted variation for further computational approaches and for the final biological interpretation, where misclassified cells can falsely introduce non-expressed genes.

In this study, we developed methods to estimate the cell population size needed for the variation of its pooled gene expression profile to equal that of bulk samples, and to detect misclassified and low-quality cells. We used the coefficient of variation (CV) together with cell population alignment strategies to measure BTM variation, and used the probability of obtaining the observed counts by sampling from the mean cell population expression to define cell divergence. These metrics enabled the identification of misclassified cells and comparison of the BTM variation among different cell populations, both for the purpose of comparing the purity of different clusters and for comparing the BTM variation between datasets to detect potential technical issues. We found that our methods were successful in detecting misclassified or low-quality cells, which were not detected by the original authors of the dataset. We also showed that PCA, UMAP, Jackstraw, and scReClassify could not detect most misclassified cells detected by DSAVE. In addition, we show that our divergence metric is consistent with other known QC metrics of low-quality cells, such as low UMI count and high mitochondrial content. Methods for cell type classification other than clustering, such as scmap [38] and CHETAH [39], could potentially be used to find misclassified cells. However, those methods rely on prior knowledge of cell types, and are currently only useful for specific cases of cell identification.

As part of this study, we developed a method to estimate the required size of a single-cell pool for the variation of its mean to be equal to that of bulk samples. Our results indicate that the required pool size is dependent on the gene expression range, where highly expressed genes require fewer cells, and vice versa. In a previous study, it was estimated that 30–100 cells were sufficient to approach the expression profile of bulk samples [40]. Although we confirmed that this holds for highly expressed genes in some datasets, the number of cells needed for lowly expressed genes to achieve the same variation as bulk is on the order of thousands.

In this work, we sought to investigate the efficacy of using cell-to-cell variation in cell populations to estimate cell homogeneity. Since the sampling noise is large and of less interest for such applications, we developed a method to estimate the non-sampling cell-to-cell (BTM) variation of a cell population such that is comparable across cell types and datasets. Our results indicate that the BTM variation is indeed useful for estimating cell homogeneity in cell populations, where removal of misclassified cells reduces the BTM variation score. The metric can thus be used for evaluating which clustering algorithm performs best for the data at hand. In addition, the BTM variation can potentially be used to detect high technical variation in cell populations. Our relative importance analysis indicates that high BTM scores relative to reference datasets is associated with certain datasets, suggesting either technical issues or cell misclassifications. Previous studies support the existence of technical variation in different forms [8, 41], which can be quantified using synthetic spike-in genes [18]. Such approaches have estimated that technical factors can explain more of the variation than their biological counterparts [42]. However, the technical noise estimated from spike-ins does not account for cell misclassifications and does not include variation introduced before the spike-ins are added (for example sample degradation), whereas these contributions are captured by our method. Although DSAVE quantifies BTM variation rather than directly measuring technical variation, our results indicate that in cases where the BTM variation is large, it is likely primarily technical.

A limitation of DSAVE is that the DSAVE variation score requires a template, which potentially will make its use more difficult if no matching template is available. Templates can technically be used across cell populations, as long as the cell populations have enough cells, enough counts per cell, and a reasonably matching set of detected genes, but it can be questioned whether the results are reliable across different types of cell populations. We show in the relative importance analysis that the BTM variation generally does not vary much across cell types when using the same template, suggesting that a template can be used across different cell types. However, the template cannot be used across species, since the gene sets would differ; new templates must therefore be created for species where no templates are available.

DSAVE is also limited in that purification of cell populations is not automated. Although DSAVE can identify which genes differ in divergent cells, it still requires manual investigation of those genes to explain why the cells are divergent. Another limitation of DSAVE is the inability to separate biological and technical contributions to the BTM variation. However, if the BTM variation is larger than what was previously observed for similar conditions, it suggests that the cause is primarily technical.

A possible future extension of the DSAVE method is to combine the identification of divergent cells with automatic cell type identification, similar to what is described in scmap [38] and CHETAH [39]. The methods could be adapted to not only detect cell types, but also other cell states such as cell cycle, expanding T cells etc. This would enable re-classification of misclassified cells as well as explain the divergence of cells that are not misclassified and motivate why they should remain in the population.

In this study, we developed the method DSAVE to improve the quality of cell populations by providing tools for a post-clustering cell population improvement step. We demonstrated that DSAVE could find misclassified cells in publicly available datasets, suggesting that DSAVE would be a useful addition to the tools currently available for analysis of single-cell data.

## Conclusions

Clustering methods are imperfect in their ability to separate cells into pure cell populations based on single-cell RNA-Seq data. DSAVE provides tools to analyze and improve the homogeneity of single-cell clusters, which supports the discovery of biological phenomena in the data. Furthermore, the tools help estimate the cell population size needed to create robust cell-type specific gene expression profiles from single-cell data. DSAVE also provides an opportunity to investigate the technical variation in datasets, which may be helpful to avoid working with publicly available datasets with high noise levels. We envision that DSAVE would make a valuable addition to the set of tools available for the analysis of single-cell RNA-Seq data.

## Supporting information

S1 Fig Evaluation of DSAVE variation score parameters. A. Correlation between the DSAVE score run with and without log transformation of data. B. Standard deviation of the DSAVE score as a function of number of repeated iterations. The score was calculated 15 times and the standard deviation of the results were plotted against the number of iterations used in each calculation. Since the standard deviation is dependent on the template, we ran the calculation for two different templates; the standard template using 2,000 cells and the modified template used in the relative importance analysis, using 1,000 cells. Fifteen iterations was selected as a reasonable balance between computation time and stability of the metric. C. Test of reproducibility for the divergence metric for a mix of B and T cells. The Pearson correlation between the two runs is 0.998, confirming that 15 iterations is enough to produce a stable metric.
(PDF)

S2 Fig Detailed investigation of the DSAVE total variation score and divergence. A. DSAVE variation score for mixed populations of T cells and monocytes as a function of the fraction of monocytes in the mix. B. Patient-to-patient variation for T cells. The figure shows the extent to which the cell-to-cell variation increases if cells from multiple patients are mixed into the cell population (equal number of cells from each patient). The datasets were aligned with a template using 1,941 cells and an average of 570 UMIs per cell to facilitate analysis of all populations. C. Relative importance of dataset, tissue and cell type for the DSAVE BTM variation score, here without the samples and covariates from the BC dataset. D. Number of detected genes per cell vs divergence for T cells from the HCA CB dataset. E. PCA showing misclassified T cells from the LC dataset detected using DSAVE. The plot shows that PCA is not able to identify the misclassified cells, at least not from the first two components. This is likely because PCA, in contrast to the divergence, looks for trends in the whole cell population; a few outlier cells will likely not have a large impact on the PCA.
(PDF)

S3 Fig Interactive divergence plot created by the DSAVE R package. The figure shows the divergence for all cells in a population of dendritic cells from the PBMC68k dataset (using cell classifications from the authors). Hovering with the mouse over a cell displays the five genes with the highest gene-wise cell divergence (i.e. the genes that diverges the most from the mean expression of the population). In this particular case we see the gene PPBP, which is a gene highly expressed in megakaryocytes, suggesting a presence of misclassified megakaryocytes in this particular cluster.
(PDF)

S1 Table Dataset access information. (PDF)

S2 Table Datasets used in figures. (XLSX)

S3 Table Genes with high variation, as discussed in S4 Note. (XLSX)

S1 Note Supplementary methods. (PDF)

S2 Note Evaluation of execution time and memory requirements. (PDF)

S3 Note Comparison with other tools for detection of misclassified cells. (PDF)

S4 Note Comparing variation across genes. (PDF)
