# Supplementary material for: DSAVE: Detection of misclassified cells in single-cell RNA-Seq data
Source: PLoS One. 2020 Dec 3;15(12):e0243360. doi: 10.1371/journal.pone.0243360 (PMC7714356; doi:10.1371/journal.pone.0243360)
Supplement: S1 Fig — A. Correlation between the DSAVE score run with and without log transformation of data. B. Standard deviation of the DSAVE score as a function of number of repeated iterations. The score was calculated 15 times and the standard deviation of the results were plotted against the number of iterations used in each calculation. Since the standard deviation is dependent on the template, we ran the calculation for two different templates; the standard template using 2,000 cells and the modified template used in the relative importance analysis, using 1,000 cells. Fifteen iterations was selected as a reasonable balance between computation time and stability of the metric. C. Test of reproducibility for the divergence metric for a mix of B and T cells. The Pearson correlation between the two runs is 0.998, confirming that 15 iterations is enough to produce a stable metric. (PDF) [file pone.0243360.s001.pdf]

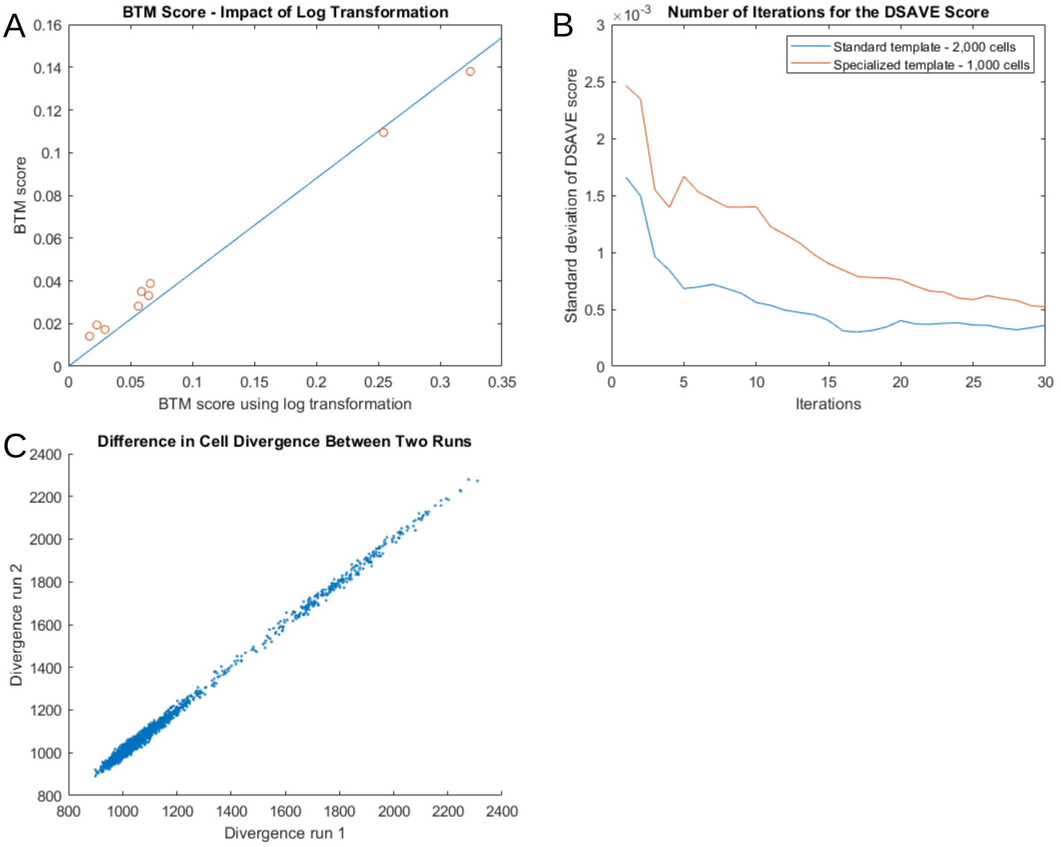

S1 Fig. Evaluation of DSAVE variation score parameters. *A. Correlation between the DSAVE score run with and without log transformation of data. B. Standard deviation of the DSAVE score as a function of number of repeated iterations. The score was calculated 15 times and the standard deviation of the results were plotted against the number of iterations used in each calculation. Since the standard deviation is dependent on the template, we ran the calculation for two different templates; the standard template using 2,000 cells and the modified template used in the relative importance analysis, using 1,000 cells. Fifteen iterations was selected as a reasonable balance between computation time and stability of the metric. C. Test of reproducibility for the divergence metric for a mix of B and T cells. The Pearson correlation between the two runs is 0.998, confirming that 15 iterations is enough to produce a stable metric.*
