# Supplementary material for: DSAVE: Detection of misclassified cells in single-cell RNA-Seq data
Source: PLoS One. 2020 Dec 3;15(12):e0243360. doi: 10.1371/journal.pone.0243360 (PMC7714356; doi:10.1371/journal.pone.0243360)
Supplement: S2 Fig — A. DSAVE variation score for mixed populations of T cells and monocytes as a function of the fraction of monocytes in the mix. B. Patient-to-patient variation for T cells. The figure shows the extent to which the cell-to-cell variation increases if cells from multiple patients are mixed into the cell population (equal number of cells from each patient). The datasets were aligned with a template using 1,941 cells and an average of 570 UMIs per cell to facilitate analysis of all populations. C. Relative importance of dataset, tissue and cell type for the DSAVE BTM variation score, here without the samples and covariates from the BC dataset. D. Number of detected genes per cell vs divergence for T cells from the HCA CB dataset. E. PCA showing misclassified T cells from the LC dataset detected using DSAVE. The plot shows that PCA is not able to identify the misclassified cells, at least not from the first two components. This is likely because PCA, in contrast to the divergence, looks for trends in the whole cell population; a few outlier cells will likely not have a large impact on the PCA. (PDF) [file pone.0243360.s002.pdf]

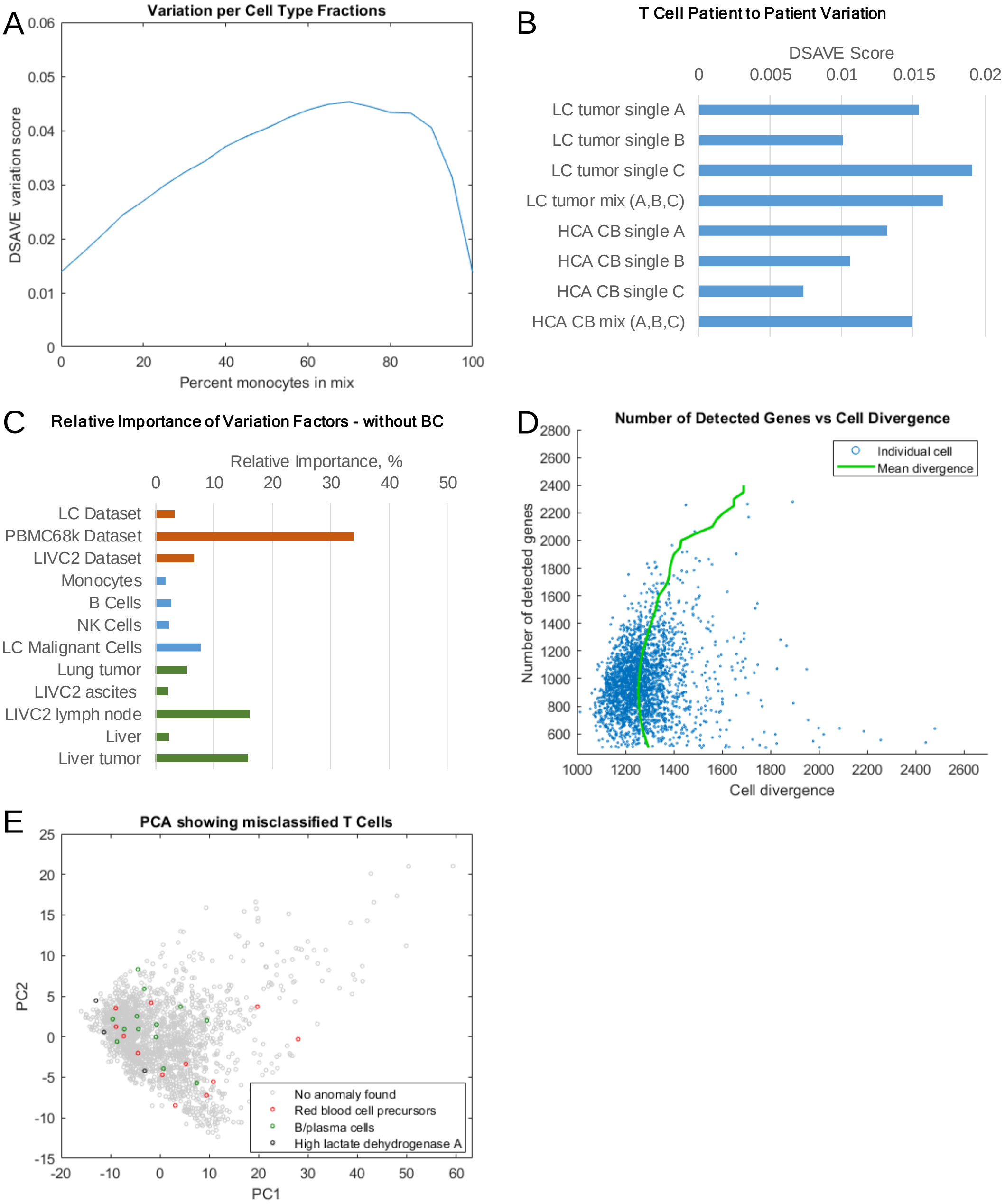

S2 Fig. Detailed investigation of the DSAVE total variation score and divergence. *A. DSAVE variation score for mixed populations of T cells and monocytes as a function of the fraction of monocytes in the mix. B. Patient-to-patient variation for T cells. The figure shows the extent to which the cell-to-cell variation increases if cells from multiple patients are mixed into the cell population (equal number of cells from each patient). The datasets were aligned with a template using 1,941 cells and an average of 570 UMIs per cell to facilitate analysis of all populations. C. Relative importance of dataset, tissue and cell type for the DSAVE BTM variation score, here without the samples and covariates from the BC dataset. D. Number of detected genes per cell vs divergence for T cells from the HCA CB dataset. E. PCA showing misclassified T cells from the LC dataset detected using DSAVE. The plot shows that PCA is not able to identify the misclassified cells, at least not from the first two components. This is likely because PCA, in contrast to the divergence, looks for trends in the whole cell population; a few outlier cells will likely not have a large impact on the PCA.*
