# Supplementary material for: DSAVE: Detection of misclassified cells in single-cell RNA-Seq data
Source: PLoS One. 2020 Dec 3;15(12):e0243360. doi: 10.1371/journal.pone.0243360 (PMC7714356; doi:10.1371/journal.pone.0243360)
Supplement: S3 Fig — The figure shows the divergence for all cells in a population of dendritic cells from the PBMC68k dataset (using cell classifications from the authors). Hovering with the mouse over a cell displays the five genes with the highest gene-wise cell divergence (i.e. the genes that diverges the most from the mean expression of the population). In this particular case we see the gene PPBP, which is a gene highly expressed in megakaryocytes, suggesting a presence of misclassified megakaryocytes in this particular cluster. (PDF) [file pone.0243360.s003.pdf]

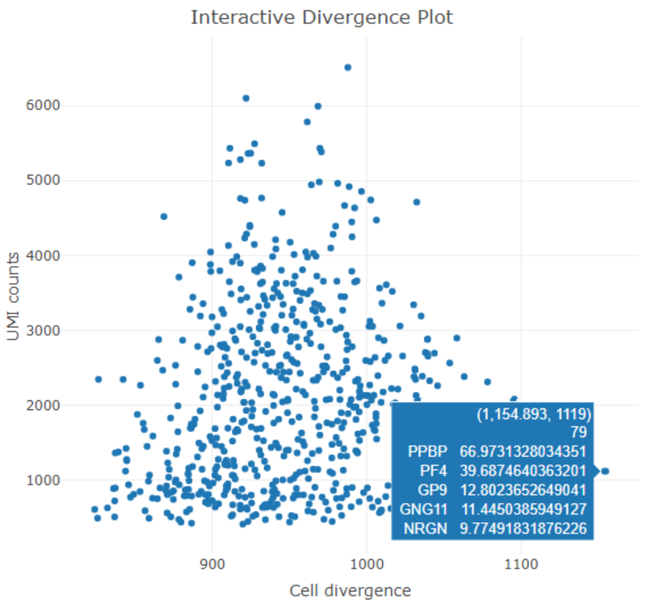

S3 Fig. Interactive divergence plot created by the DSAVE R package. *The figure shows the divergence for all cells in a population of dendritic cells from the PBMC68k dataset (using cell classifications from the authors). Hovering with the mouse over a cell displays the five genes with the highest gene-wise cell divergence (i.e. the genes that diverges the most from the mean expression of the population). In this particular case we see the gene PPBP, which is a gene highly expressed in megakaryocytes, suggesting a presence of misclassified megakaryocytes in this particular cluster.*
