# Supplementary material for: DSAVE: Detection of misclassified cells in single-cell RNA-Seq data
Source: PLoS One. 2020 Dec 3;15(12):e0243360. doi: 10.1371/journal.pone.0243360 (PMC7714356; doi:10.1371/journal.pone.0243360)
Supplement: S1 Table — (PDF) [file pone.0243360.s004.pdf]

S1 Table. Dataset access information

ID	Source
HCA CB	The data can be downloaded from https://data.humancellatlas.org/, Census of immune cells.
BC	The data is available for download on GEO data repository, accession numbers: GSE114727, GSE114725, and GSE114725.
LC	The data is available in in ArrayExpress under accessions E-MTAB-6149 and E-MTAB-6653.
OC	The dataset is published at figshare together with the associated code.
LIVC	The data is available for download on GEO data repository, accession number GSE98638.
PBMC68k	The data is available at 10x Genomics' home page.
B10k	The data is available at 10x Genomics' home page.
CD4TMEM	The data is available at 10x Genomics' home page.
TCD8	The data is available for download on GEO data repository, accession number GSE 112845.
LIVC2	The data is available for download on GEO data repository, accession number GSE 140228.
BLUEPRINT data	The data can be downloaded from the BLUEPRINT Epigenome Project. The samples were taken from the project EGAD00001001173 and have the following sample IDs: S002EV11, S004M711, S007DD11, S007G711, S008H111, S009W411, S001FRB1, and S0041C11 EGAD00001001173. http://www.blueprint-epigenome.eu/
