# Supplementary material for: DSAVE: Detection of misclassified cells in single-cell RNA-Seq data
Source: PLoS One. 2020 Dec 3;15(12):e0243360. doi: 10.1371/journal.pone.0243360 (PMC7714356; doi:10.1371/journal.pone.0243360)
Supplement: S1 Note — (PDF) [file pone.0243360.s007.pdf]

DSAVE: Detection of misclassified cells in single-cell RNA-seq data

S1 Note – Supplementary methods

Overview

This note contains additional details about the DSAVE method to what is presented in the main text.

Definition of Variation

To better understand BTM variation, we provide an alternative explanation of the different types of variation used in the main text. 1) We define sampling noise as the variation caused by the limited number of molecules that are detected for each cell, which provide only a sample of the true gene expression distribution. This gives rise to a discrete probability distribution with limited resolution. 2) The BTM variation, including all variation except sampling noise, can be viewed as the variation in the probability distributions from which reads are sampled.

Additional advice for DSAVE users

As described in the main text, the DSAVE BTM score and cell divergence operates on cell subpopulations, where the cells are typically expected to belong to a certain cell type. The divergence metric of a cell is a comparison against the mean expression of the cell population. It is therefore recommended to remove low quality cells, as identified for example by too few UMI counts or high mitochondrial content, before analysis. Such cells would otherwise be included in the cell populations and could potentially affect the average expression of the population, as well as contribute to the total computation time of the analysis.

A potential method to use if the cell population is large is to run the divergence analysis on a smaller subset of the cells, and to identify by cell markers what type of misclassified cells exist in the cluster. Such a method is expected to remove a large portion of the misclassified cells with a substantial reduction in computational time.

If the cell population contains few UMIs per cell and the BTM calculation cannot be calculated using a certain template, it may be helpful to remove cells with low number of UMIs per cell, which will increase the average UMI counts per cell.

It only takes a few minutes to investigate a cell population from a dataset using the DSAVE BTM score, and we argue that this is time worth spending. If the score is much higher than that of most other cell populations (such as in the BC dataset), we have shown that the variation is likely not biological, and in such cases the data will likely be difficult to work with unless this is corrected. This can in many cases be solved by improving the clustering and/or filtering out more cells. It could also be that additional methods, such as more advanced normalization, could be applied to reduce the effect in case it is technical.

DSAVE total cell pool variation estimation

It should be noted that the Total Cell Pool Variation Estimation differs from other DSAVE metrics, which are based on the coefficient of variation (CV). This was to ensure a fair comparison of the variation between single-cell pools and bulk samples. A CV calculation requires the assembly of many cell pools, which requires sampling cells with replacement unless the cell population is very large. Sampling single cells with replacement when calculating the Total Cell Pool Variation Estimation would artificially reduce variation relative to bulk profiles, as the latter is not comprised of any duplicate cell profiles.

In addition, the algorithm takes a gene expression range as input, where genes with a mean expression over all cells that is outside the range are excluded. In general, a narrower range (e.g., 0.5

– 2 TPM/CPM) provides a fairer comparison, but will then be representative for only that range. A large range may provide an overall view of the variation, but since the proportion of highly vs. lowly expressed genes may differ between samples, and lowly expressed genes tend to exhibit higher variation, this may bias the results. A possible extension of this metric would be to work on an average over gene expression ranges instead of the mean over all genes.

DSAVE BTM variation score

Cell population alignment and template generation

Cell population alignment uses downsampling to increase the sampling noise to the same level for all cell populations. This can be justified by the following reasoning: downsampling the number of counts per cell will only introduce more sampling noise, since randomly discarding reads is equivalent to having fewer reads, and will thus impart minimal changes to the BTM variation.

The alignment procedure includes a downsampling step to match the sampling noise to that stated by the template. To accomplish this, the template contains a vector $\mathbf{n_t}$ of total counts per cell for the same number of cells as should be included in the aligned cell population. A vector $\mathbf{n_c}$ representing the total counts per cell in the population to be aligned is also calculated. The vectors $\mathbf{n_c}$ and $\mathbf{n_t}$ are then sorted. A vector $\mathbf{n_d}$ representing the number of counts to remove from each cell is then calculated as

$$\mathbf{n_d} = \mathbf{n_c} - \mathbf{n_t}$$

The cells are then downsampled with the number of counts stated in $\mathbf{n_d}$, which means that the number of counts to remove stated in $\mathbf{n_d}$ for cell c, $n_{d,c}$, are randomly removed for that cell in the count matrix. If any negative values exist in $\mathbf{n_d}$, removing that amount of counts would practically mean adding of counts. This is not possible in a downsampling procedure. Instead of adding counts to the cells, this is compensated for by having less counts removed from other cells in the downsampling procedure. This compensation normally only occurs for few counts in total and is of little importance for the variation, and although it could theoretically introduce a bias it makes it possible to use the template on more datasets.

The standard template that is provided as part of DSAVE is designed for use with human data and is generated from the T cells in sample BC4_TUMOR from the BC dataset. Included genes were selected as the intersection of the genes in the datasets BC, OC, B10k and TCD8. The number of cells and the average number of UMIs per cell were set to 2000 and 750, respectively, and the fraction of upper and lower outlier genes was set to 0.025 (for both values). This template was used throughout this study unless otherwise stated. Increasing the number of cells or the number of UMIs per cell will improve the precision of the metric, which may motivate the use of alternative templates, and reducing those metrics would instead make it useful for more datasets.

Calculation of variation

The DSAVE variation metric is based on the coefficient of variation (CV), calculated gene-wise over all cells in the population. DSAVE supports two alternatives for calculating the variation; logarithm of CV (described in the main text), and CV of log-transformed data, where the latter is less impacted by outliers. The alternatives correlate well with each other (**Error! Reference source not found.**E). In this study, all figures and results were generated using the logarithm of CV.

When using the CV of log-transformed data, the CPM/TPM expression is first transformed to $E_{tr} = ln(E_{CPM} + a)$, where E_{CPM} is the count value transformed to CPM/TPM and a is a parameter that we have set to 1 to yield a reasonable variation in D_i for a gene across the gene expression range and to keep all values positive. D_i is then calculated as $D_i = (SD_{lt,i})/(M_{lt,i} + 0.05)$, where $SD_{lt,I}$ and $M_{lt,i}$ are the standard deviation and mean of E_{tr}. The factor 0.05 is to avoid dividing by a very small number or zero, minimizing the impact of very lowly expressed genes. The value 0.05 was selected empirically to yield a reasonably variable BTM variation over the gene expression range.

To reduce the effect of outlier genes on the variation score, we remove a percentage of genes with the highest and lowest variation. These cutoffs are parameters of the template and were set to 2.5% for both the low and high cutoff; the parameter values were empirically selected as a balance between discarding the worst outliers while retaining the variation in the cell populations.

Software

DSAVE is implemented in both MATLAB and R to increase its availability.

The MATLAB code and all code for reproducing the figures is available at GitHub (github.com/SysBioChalmers/DSAVE-M). For installation, first clone the repository (or download the zip and extract). Open MATLAB and set the current directory to the repository root. Run the command “DSAVEInstall.install();”. The code is now ready for use. If the user desires to reproduce the figures in this paper, additional data is required. The data originates from public datasets, but may be cumbersome to assemble, since 9 single-cell datasets are used. Advice on how to assemble the data can be given upon reasonable request. Some of the figures were generated in Microsoft Excel from data produced with MATLAB and R.

DSAVE is also available as an R package on GitHub (github.com/SysBioChalmers/DSAVE-R). Installation instructions are available at the GitHub page.
