# Supplementary material for: DSAVE: Detection of misclassified cells in single-cell RNA-Seq data
Source: PLoS One. 2020 Dec 3;15(12):e0243360. doi: 10.1371/journal.pone.0243360 (PMC7714356; doi:10.1371/journal.pone.0243360)
Supplement: S2 Note — (PDF) [file pone.0243360.s008.pdf]

DSAVE: Detection of misclassified cells in single-cell RNA-seq data

S2 Note – Evaluation of execution time and memory requirements

Overview

This note contains an evaluation of the expected run time and memory usage for DSAVE.

Computational limits and performance

Single-cell RNA-Seq datasets continue to grow in size; it is therefore of interest to know how well DSAVE performs for large datasets. DSAVE supports three major calculations; 1) estimation of pool size to achieve the same variation as in bulk, 2) the BTM variation score, and 3) the single-cell divergence. We have analyzed the big-O complexity of the software and provided performance measurements for the second and third calculation.

We estimate the execution time of the first calculation to grow linearly with the pool size tested, as it relies on sampling of cells and calculating CVs over those cells (which both have linear complexity). It is in most cases not relevant to test this for pool sizes larger than a few thousand (where the curve flattens out), which is what is shown in the package vignette, and this operation typically finishes within a few minutes on a normal desktop computer. The performance should in theory be independent of total cell population size, but since the current implementation relies on converting the sparse matrix supplied to a dense matrix, we recommend subsetting the data matrix provided for the calculation to around 10,000 cells to avoid large memory allocation. The calculation has good potential to fully support sparse matrices in future releases as well as have calculations performed in parallel, since many independent iterations are performed.

The BTM score calculation is virtually independent of total cell population size since it randomly samples a cell subset of the data, where the number of cells is set in the template, before performing any calculations. We estimate the calculation time to grow linearly with the number of cells stated in the template, since the calculation is based on downsampling and CV calculations. We performed a test run that indicates that the execution time is independent of total cell population size (Fig A I), and grows approximately linearly with template size (Fig A II). In additional, we calculated the BTM variation for 68,000 cells using a template with 2,000 cells, which took 419 seconds. The memory consumption grows linearly with the template size, which is usually within a few thousand cells and something a normal desktop computer can easily handle. The limitation of cell population size is therefore mainly dependent on the sparse data matrix fitting within memory. The calculation has good potential to be parallelized in future releases since many independent iterations are performed. For very large cell populations, where memory can be an issue, the BTM score can be calculated from a randomly selected subset of the population. If several batches of data exist, the calculation can be run per batch, which could also provide information about differences in variation across batches.

The cell divergence metric calculation grows linearly with the number of cells. It furthermore grows at a maximum linearly with the number of genes, the target number of UMIs to which the cell is downsampled, and the average original number of UMI counts per cell. We performed a test run which indicates that the execution time grows slightly more than linearly with the number of cells, most likely depending on that more cells will also include more genes, leading to more data to handle (Fig A III). The memory consumption is small, since the cells are processed individually, which also makes the algorithm parallelizable. As in the case with the DSAVE BTM score, the memory limitation on cell population size is therefore mainly dependent on that the sparse data matrix must fit in memory. For very large cell populations, where memory can be an issue, the cell populations can be split up into smaller subpopulations and be run separately, which will likely only have minor effect on the calculations (the mean expression of the population may vary slightly). Furthermore, if

the purpose of the divergence calculation is to identify types of divergent cells, such cells can usually be identified from a cell subpopulation, which can save considerable computation time.

The tests were run on a HP Elitebook 840 G3 running Windows 10 with 4 cores and 16 GB of RAM. For such a computer, our tests suggest that DSAVE can easily handle cell populations of 10,000 cells, and most likely more since the major limitation is the memory allocated by the sparse matrix in the dataset. It is also worth noting that cell populations of 10,000 cells corresponds to much larger datasets, since it is common to at least have 5 to 10 different cell populations in a single-cell dataset.

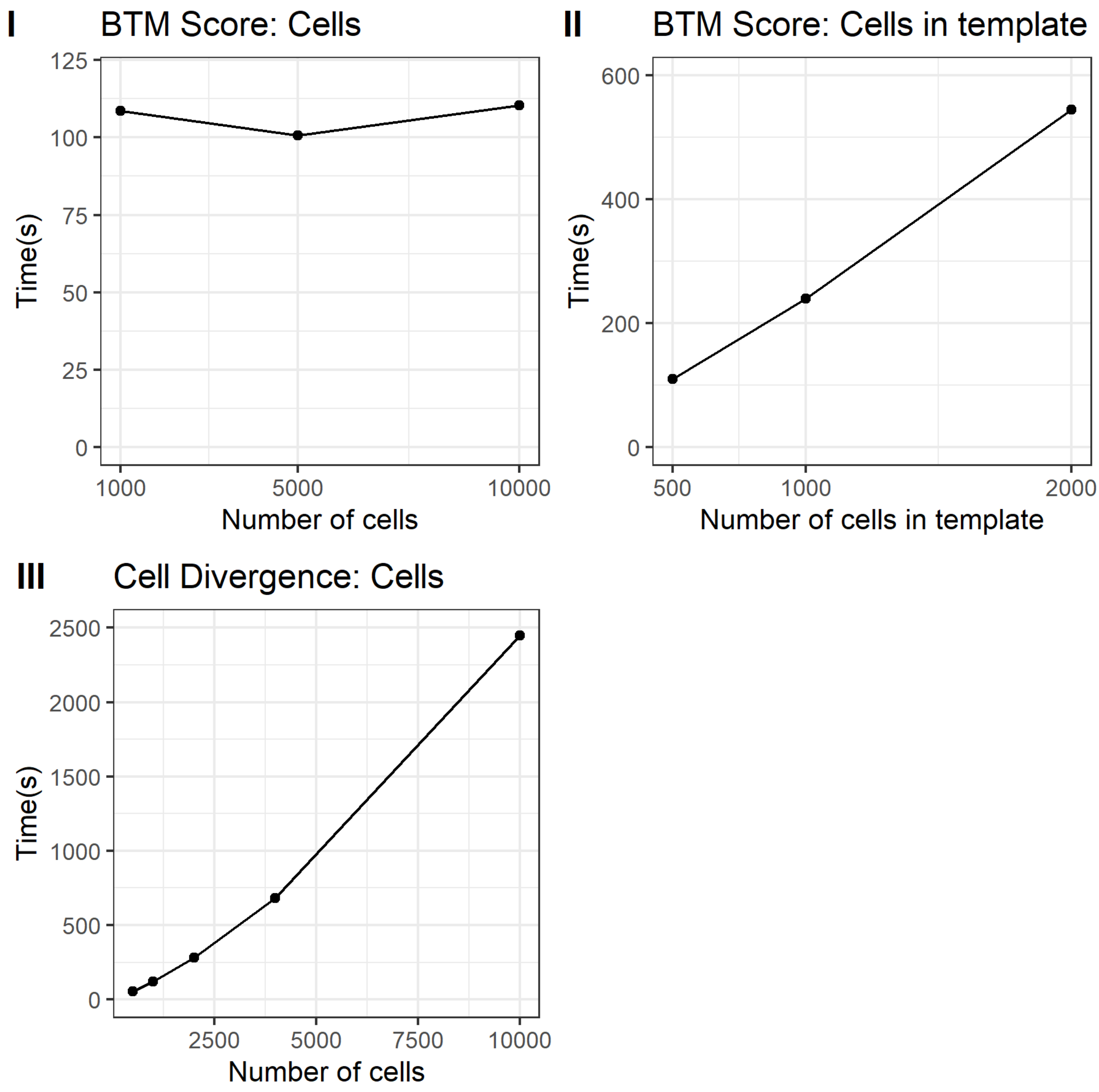

Fig A. DSAVE performance analysis. *I. Execution time vs number of cells for the BTM score calculation. II. Execution time vs number of cells included in the template for the BTM score calculation. III. Execution time vs number of cells for the cell divergence calculation. The execution times presented for all plots is the average time out of 10 runs.*
