# Supplementary material for: DSAVE: Detection of misclassified cells in single-cell RNA-Seq data
Source: PLoS One. 2020 Dec 3;15(12):e0243360. doi: 10.1371/journal.pone.0243360 (PMC7714356; doi:10.1371/journal.pone.0243360)
Supplement: S3 Note — (PDF) [file pone.0243360.s009.pdf]

DSAVE: Detection of misclassified cells in single-cell RNA-seq data

S3 Note – Comparison with other tools for detection of misclassified cells

Overview

This note contains a comparison of DSAVE with Jackstraw and scReClassify in terms of their ability to find misclassified cells.

Comparison between tools about the ability to find misclassified cells

It is generally difficult to compare the performance of methods detecting misclassified cells, since a ground truth is not available. DSAVE is a tool which helps the user to manually find divergent cells; it is therefore not meaningful to directly compare the cells identified with DSAVE to the cells identified with the other tools, since it depends on how much effort the user chooses to spend on purification using DSAVE. We therefore focused on investigating whether the other tools could find a group of misclassified cells identified using DSAVE, where we focused on divergent cells with highly divergent genes usually expressed in NK-cells and/or cytotoxic T-cells (GNLY, GZMB, NKG7) in a cluster containing mainly FCGR3A+ monocytes.

We used the 20,000 first cells in the PBMC68k dataset for the analysis and analyzed them using Seurat (v. 3.1.1). We used k-means clustering (stats R package, version 3.6.1) instead of the built-in clustering method in Seurat, since this simplified the use of Jackstraw. The clusters were visualized in UMAP space (Fig A), and we chose to investigate cluster 4 since all cells from that cluster were clustered together in UMAP space (for some clusters the kmeans clustering contained cells widely spread across UMAP space, which leaves a lot of obvious misclassifications). We plotted cell type markers in UMAP space to determine the cell type of cluster 4 (Fig B) and concluded that the cluster likely mainly contains FCGR3A+ monocytes (due to the markers FCGR3A and MS4A7).

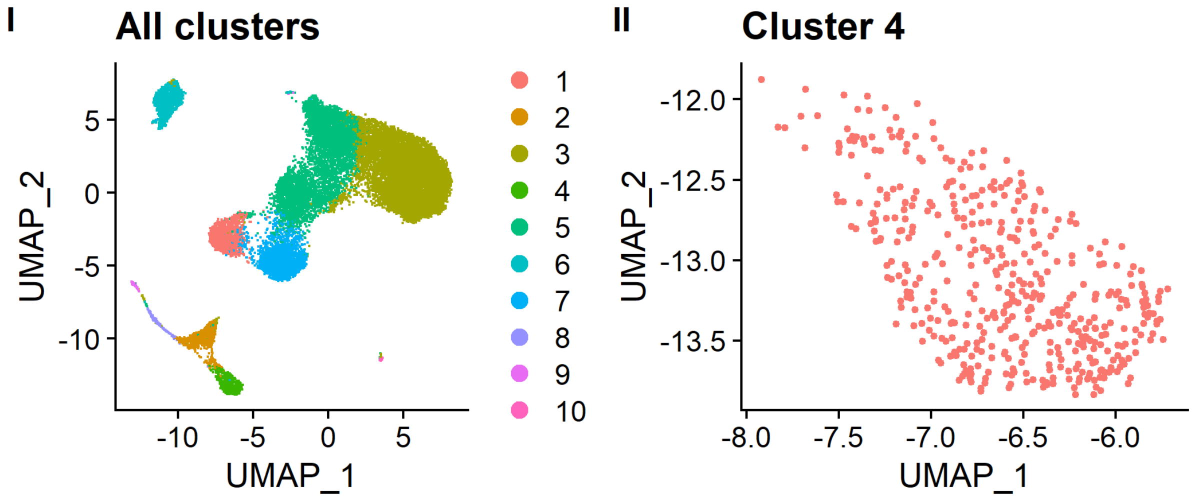

Fig A. Clustering displayed in UMAP space. *I. All cells colored per cluster. II. The cells from cluster 4.*

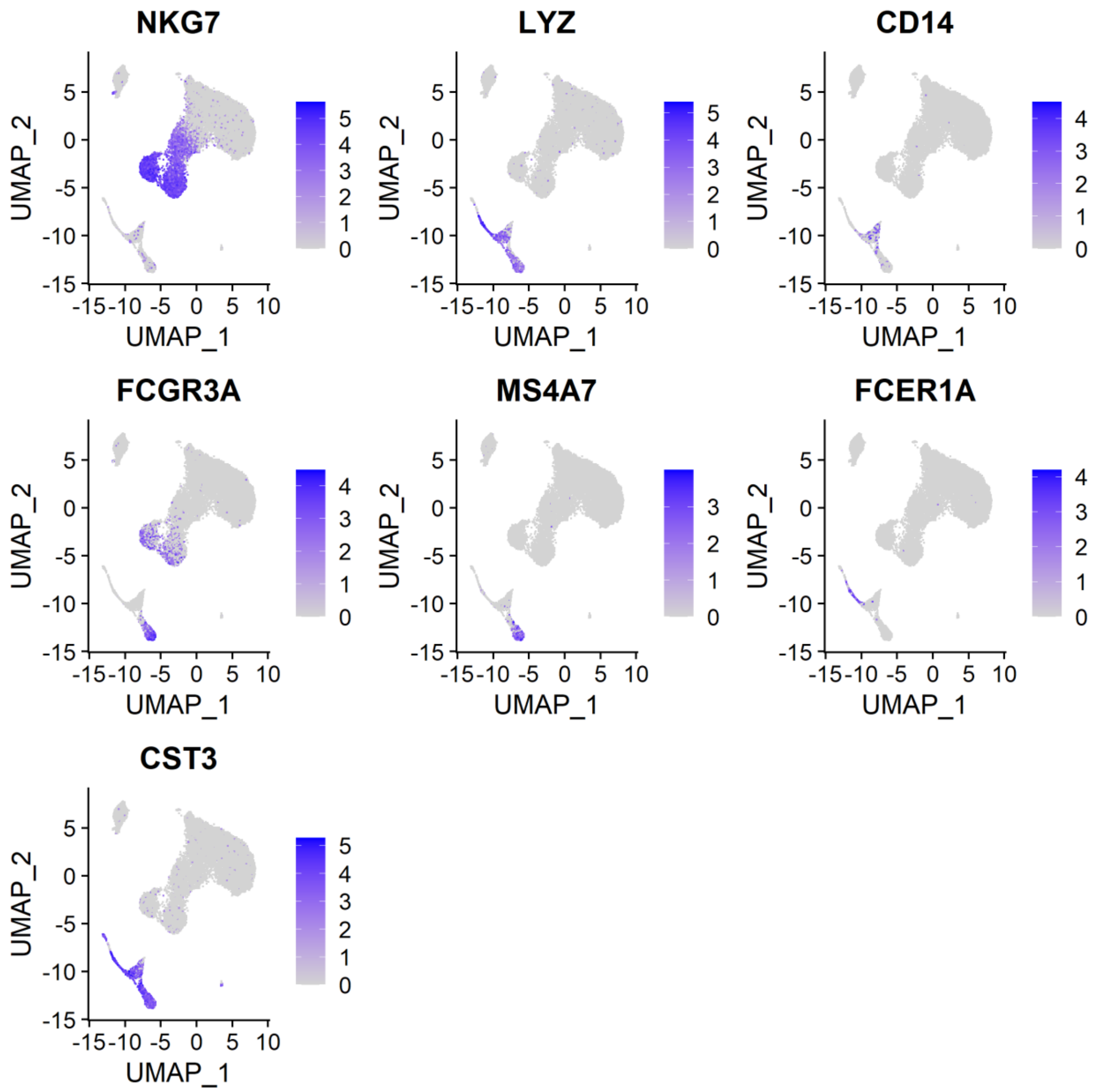

Fig B. Expression of cell type markers. *Cluster 4, positioned at the bottom, has high expression of FCGR3A and MS4A7, suggesting FCGR3A+ monocytes.*

The DSAVE divergence was then calculated, followed by examination of divergent cells. 7 cells in cluster 4 (containing in total 463 cells) with high divergence had a high presence of NK-cell and/or cytotoxic T cell markers (GNLY, GZMB, NKG7), suggesting these cells to be either misclassified cells or doublets. We chose to focus on those cells to see if they could be identified by the other tools. Jackstraw can be used in a variety of ways, for example with different dimensionality reduction methods. We applied Jackstraw (version 1.3) with PCA (10 first PCs), and defined misclassified cells as having a posterior inclusion probability (PIP) value of less than 0.8, as described in the package vignette (https://htmlpreview.github.io/?https://github.com/ncchung/jackstraw/blob/master/vignettes/jackstraw-cluster.html). Jackstraw found 14 cells with PIP below 0.8, of which one (the most divergent one in DSAVE) overlapped with the seven selected cells (Fig C). We also applied scReClassify (version 0.1.0) on all cells, supplying it with 20 PCs. In total, scReClassify reclassified 312 cells, and we deem it likely that scReClassify corrected much of the shortcomings of the kmeans clustering. However, scReClassify did not reclassify a single cell in cluster 4. We conclude that although these tools likely

help with detecting misclassified cells, DSAVE can be used to find misclassified cells not found by these methods, at least not with the parameterization used in this evaluation. We note that we in this analysis focused on the NK cells and did not investigate cause of divergence for the other cells.

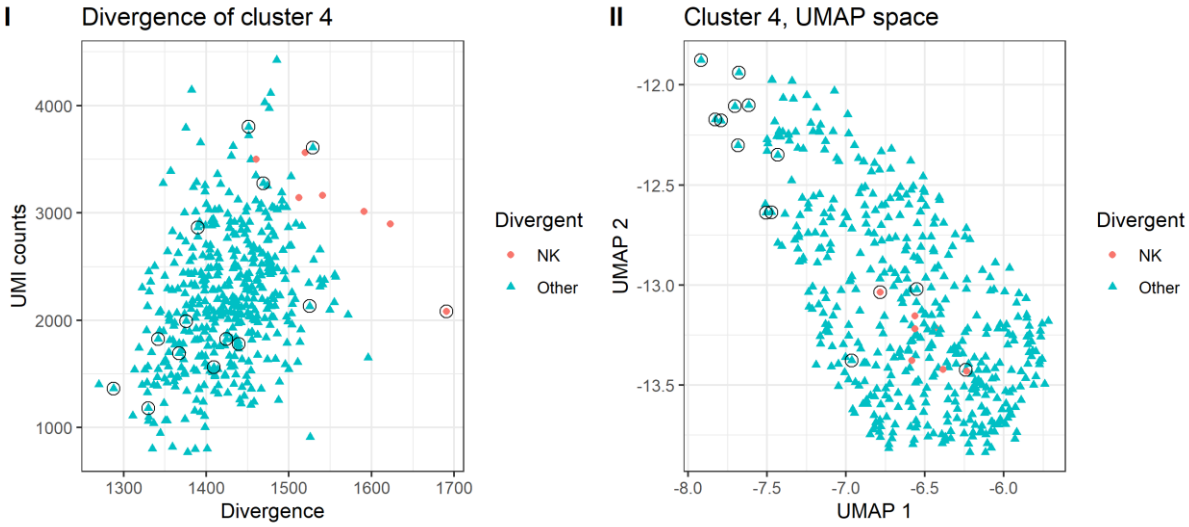

Fig C. Overlap of detected misclassifications between DSAVE and Jackstraw. *The cells detected by Jackstraw are encircled in black. I Misclassified cells displayed in UMI counts vs DSAVE divergence space. II. Misclassified cells displayed in UMAP space.*
