# Supplementary material for: DSAVE: Detection of misclassified cells in single-cell RNA-Seq data
Source: PLoS One. 2020 Dec 3;15(12):e0243360. doi: 10.1371/journal.pone.0243360 (PMC7714356; doi:10.1371/journal.pone.0243360)
Supplement: S4 Note — (PDF) [file pone.0243360.s010.pdf]

DSAVE: Detection of misclassified cells in single-cell RNA-seq data

S4 Note – Comparing variation across genes

Overview

This note contains a description of an additional feature in DSAVE which can be used to compare BTM variation across genes.

DSAVE gene-wise variation metric

In addition to the metrics described in the main text, DSAVE also provides a gene-wise metric for determining which genes exhibit the most BTM variation in a population. The metric is similar to the DSAVE variation score but is calculated individually for each gene. As part of the calculation, sampling noise only (SNO) cell populations are generated in a similar way as for the DSAVE variation score, but instead of requiring each *cell* in the SNO cell population to have the same number of counts as in the original cell population, each *gene* is required to have the same number of counts across all cells in the SNO population as in the original. When simulating counts to generate the SNO population, the probability of assigning a count to a gene in a given cell is proportional to the total number of counts for that cell in the original cell population. This ensures a similar distribution of total counts per cell between the SNO and original cell populations. The original dataset is not aligned in any way, meaning that the gene-wise variation metric is not comparable between cell populations. This is motivated by that we do not want to discard any reads due to down-sampling, since that would make the variation less reliable.

We also calculate p-values indicating whether the variation for a particular gene is greater than what would be expected by chance. The p-value calculation is based on a non-parametric test since it was difficult to fit the data to a known distribution. The p-value for a certain variation value D_i for gene i is then calculated as the fraction of SNO variation values of the same gene that is larger or equal to D_i.

SNO generation is repeated multiple times to reduce stochasticity. The number of repetitions is a parameter that can be selected depending on the purpose of the analysis. For calculating the BTM variation, 100 repetitions is sufficient, while we recommend 10,000 – 100,000 repetitions for calculating p values, which is motivated by Fig B below.

Different genes show large differences in BTM variation

We estimated the BTM variation for individual genes to investigate the difference in BTM variation between genes, and to determine if the DSAVE variation score is dominated by a few outlier genes. Fig A I shows the distribution of gene-wise BTM variation among all genes with a mean expression above 1 CPM for three T cell populations of 2,000 cells each from different datasets. In general, there is a large difference in BTM variation between genes.

Stochasticity is of little concern when evaluating the overall BTM variation for a cell population because the result is an average over many genes. For single genes, however, there is a risk that the BTM variation for a gene is the result of sampling stochasticity, since the BTM variation is calculated from the difference between the total variation and that of the average sampling noise. To assess this risk, we generated SNO cell populations in such a way as to preserve the average gene expression for each gene and measured the total variation individually per gene. Repeating this process yielded a distribution of total variations from which we could draw inference. Fig A II shows the 95% one-sided confidence interval of the magnitude of the BTM variation as a function of gene expression for a cell population of 2,000 cells, under the null hypothesis that the total variation for that gene is only sampling noise. In general, the BTM variation needs to be higher for lowly expressed genes to suggest non-negligible BTM variation. The breast cancer T cell population contained the most genes (6,653) with significant BTM variation (using the Benjamini-Hochberg correction for multiple testing with an FDR of 0.05, only testing genes with CPM ≥ 1). The

corresponding number of genes for the lung cancer and HCA cord blood T cell populations were 2,137 and 1,050, respectively.

The gene-wise metric itself is not comparable between cell populations, but we were interested in the intersection among the highly variable genes across different datasets to determine if any general conclusions could be drawn for certain genes. Fig A III shows the intersection of the 250 most variable genes (with significant BTM variation) from three datasets, where some genes are highly variable in more than one cell population. The small overlap despite having the same cell type suggests that the dominating factors for the gene variation are either technical or related to tissue differences. The most variable genes from the three cell populations are listed in Table S3, where STMN1 was present as highly variable in all datasets. We also observed that the genes HBA1, HBA2 and HBB were among the most highly variable genes in both the LC and BC datasets. In the main text, we show that some cells in the LC T cell population are likely red blood cell precursors, which is consistent with this finding, and it seems such cells are also present in the BC T cell population.

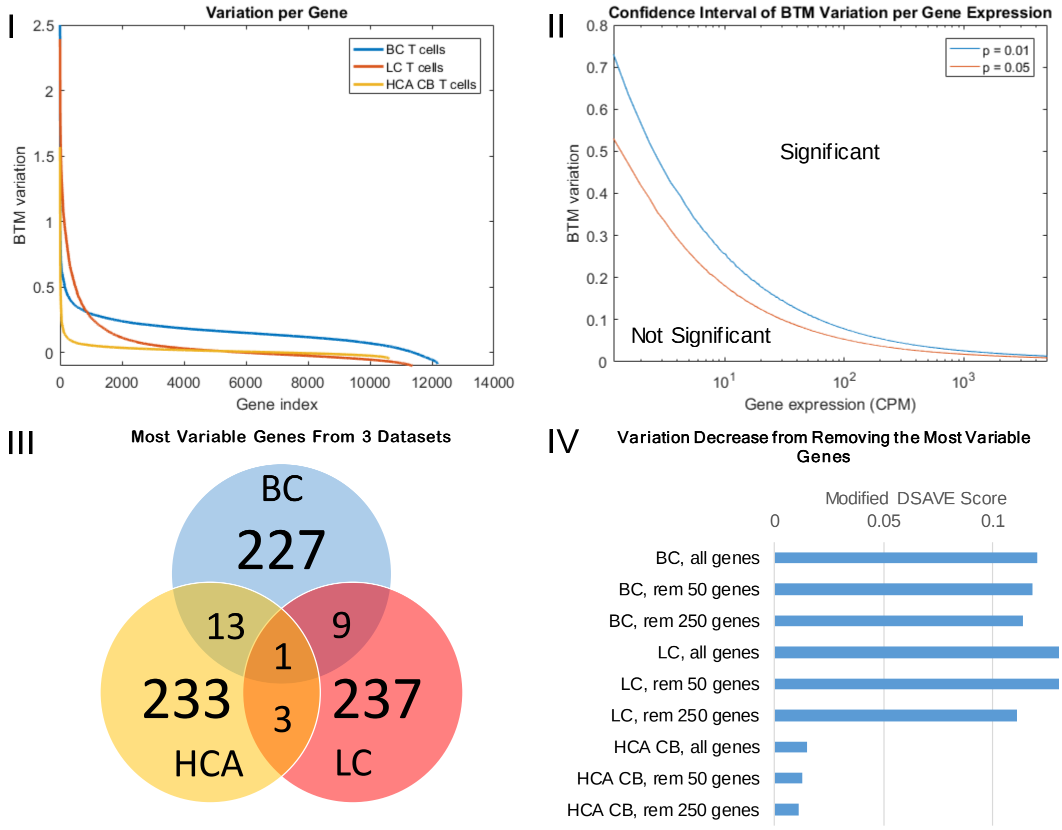

Fig A. Evaluation of the gene-wise variation metric. *I. Gene-wise BTM variation for T cells from 3 datasets. The genes have been sorted by descending BTM variation. Only genes with CPM ≥ 1 are shown. The breast cancer and lung cancer cells originate from a mix of patients, whereas the Human Cell Atlas cord blood T cells are from a single patient. II. The 95% and 99% confidence intervals indicating whether the gene-wise BTM variation is higher than the sampling noise. III. Venn diagram showing the overlap between the 250 most variable genes across the T cells from the three datasets in panel I. IV. Variation decrease caused by replacing the most variable genes with only sampling noise for T cells from 3 datasets. The effect is clear but not dramatic, suggesting that the most variable genes do not completely dominate the DSAVE variation score. The DSAVE variation score template was modified here to not remove any outliers.*

To analyze the impact of the most variable genes on the DSAVE variation score, we replaced the counts for those genes with simulated counts from a matching SNO dataset. Fig A IV shows the effect of replacing the 50 or 250 most variable genes. There is a clear effect, however not dramatic, and the effect appears to be similar between datasets. This suggests that replacing the most variable genes from the DSAVE score will yield a score that is more representative of most genes. We note, that the LC dataset here gives a much higher BTM variation score than what is presented in the main

text. The data used here is a subset of the T cells (the 2000 first cells in the matrix), that are seemingly not representative for the dataset as a whole, which suggests that those cells may be of lower quality than the rest of the cells in the dataset.

Evaluation of the difference in variation between genes

Since the gene-wise metric is computationally expensive, it is helpful to determine how many iterations are needed to obtain a stable value, both for the metric itself and the associated p-values. We repeated the gene-wise measurements twice for the same data and same number of iterations. Fig B I shows the correlation per gene between two runs for the BTM gene-wise variation metric at 100 iterations. The corresponding Pearson correlation is 0.9991, indicating that 100 iterations is enough to achieve stable values for the metric itself. For the p values, more iterations are required. Fig B II-IV shows the correlation of the p values for two runs at 100,000, 10,000, and 1,000 iterations, respectively. The corresponding Pearson correlations are 0.9995, 0.9960, and 0.9644 (p-values larger than 0.05 excluded); from this we recommend at least 10,000 iterations to acquire stable p values.

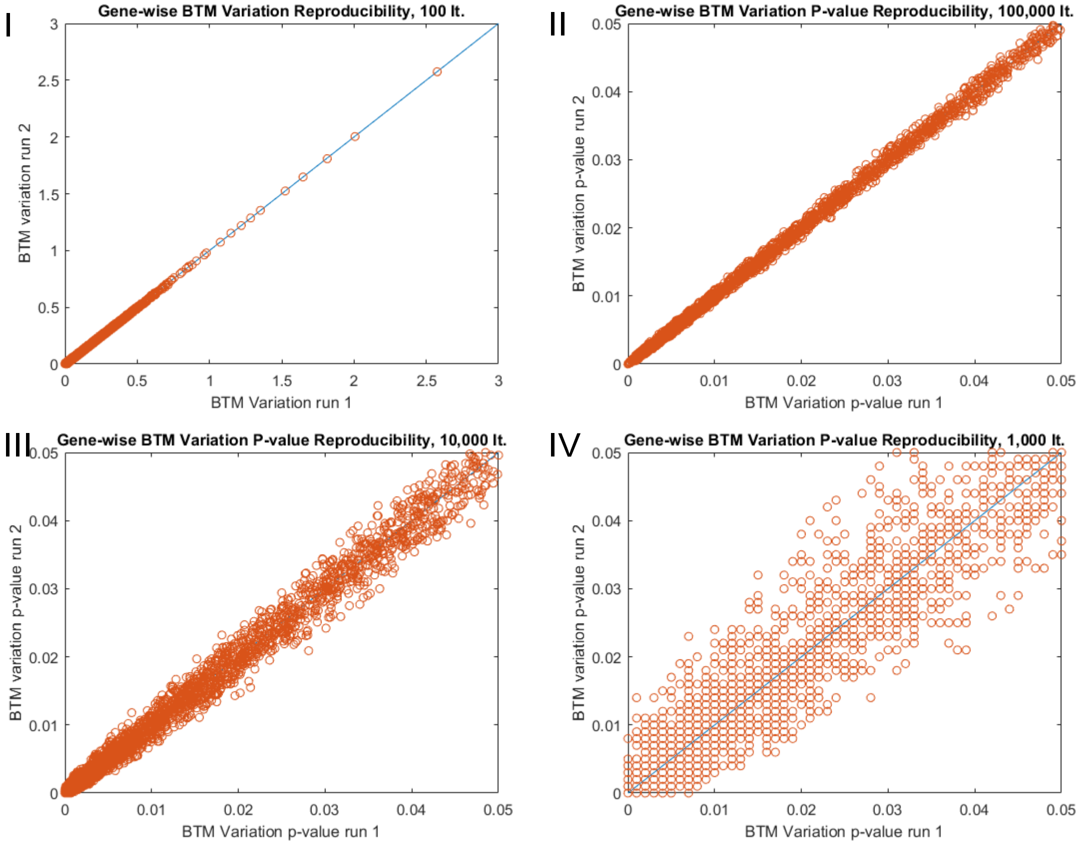

Fig B. Reproducibility for the DSAVE gene-wise metric. *(I) Correlation for the BTM variation of genes between two runs with 100 iterations. (II, III and IV) Correlation for the p-values of the BTM variation of genes between two runs for 100,000, 10,000 and 1,000 iterations, respectively.*
